# A Case Study of Eukaryogenesis: The Evolution of Photoreception by Photolyase/Cryptochrome Proteins

**DOI:** 10.1007/s00239-020-09965-x

**Published:** 2020-09-26

**Authors:** Jennifer A. Miles, Thomas A. Davies, Robert D. Hayman, Georgia Lorenzen, Jamie Taylor, Mubeena Anjarwalla, Sammie J. R. Allen, John W. D. Graham, Paul C. Taylor

**Affiliations:** 1 School of Chemistry, University of Leeds, Woodhouse Lane, Leeds, LS2 9JT UK; 2 Astbury Centre for Structural Molecular Biology, University of Leeds, Woodhouse Lane, Leeds, LS2 9JT UK

**Keywords:** Cryptochrome, Photolyase, Cyanobacteria, Horizontal gene transfer, Eukaryogenesis

## Abstract

Eukaryogenesis, the origin of the eukaryotes, is still poorly understood. Herein, we show how a detailed all-kingdom phylogenetic analysis overlaid with a map of key biochemical features can provide valuable clues. The photolyase/cryptochrome family of proteins are well known to repair DNA in response to potentially harmful effects of sunlight and to entrain circadian rhythms. Phylogenetic analysis of photolyase/cryptochrome protein sequences from a wide range of prokaryotes and eukaryotes points to a number of horizontal gene transfer events between ancestral bacteria and ancestral eukaryotes. Previous experimental research has characterised patterns of tryptophan residues in these proteins that are important for photoreception, specifically a tryptophan dyad, a canonical tryptophan triad, an alternative tryptophan triad, a tryptophan tetrad and an alternative tetrad. Our results suggest that the spread of the different triad and tetrad motifs across the kingdoms of life accompanied the putative horizontal gene transfers and is consistent with multiple bacterial contributions to eukaryogenesis.

**Electronic supplementary material:**

The online version of this article (10.1007/s00239-020-09965-x) contains supplementary material, which is available to authorized users.

## Introduction

It is widely accepted that eukaryogenesis involved an endosymbiosis between at least one archaeon, probably from the Asgard lineage, and at least one bacterium, most likely an ancestral *α*-proteobacterium (López-García and Moreira 2019). More controversial and less well understood is the possibility of further symbioses or other significant gene flows from bacteria to ancestral eukaryotes (Pittis and Gabaldón 2016; Spang et al. 2019). Both the order in which these key evolutionary events occurred and their timeline are unclear. Characterising the emergence of animals from this ancient mix is of particular interest and offers a useful perspective from which to explore the major transitions (Paps 2018).

In addition to the large-scale phylogenomic studies that underpin our emerging understanding of eukaryogenesis, the field may benefit from case studies of the evolution of individual genes/proteins across the kingdoms of life. Researchers are finding surprising similarities in chemical signalling-related proteins between animals and groups of complex bacteria, including cyanobacteria (Brash et al. 2014; Magnani et al. 2017; Miles et al. 2019; Millard et al. 2014; Picciano and Crane 2019; Ponting et al. 1999; Rawlings 2015) that pose some important questions about horizontal gene transfer (HGT) in the origin of animals. This offers an alternative perspective from which to view the major transitions in eukaryogenesis. A good object of study would therefore be a family of proteins found in many major kingdoms and taxa, including animals, and that possesses a highly distinctive biochemical motif or motifs that would allow us to further refine phylogenetic analyses.

The photolyase/cryptochrome superfamily is distinguished biochemically by the ability of these proteins to harbour radical-pair intermediates (Hore and Mouritsen 2016). This phenomenon allows photolyases and cryptochromes to sense and react to sunlight, which is of enormous benefit to many species. There are two broad types of response, namely DNA repair and signalling (Mei and Dvornyk 2015). In particular, ultraviolet B (UVB) present in sunlight can be damaging to genetic material, changing the chemical structure of DNA, leading to aberrations such as cyclic pyrimidine dimers (CPDs) and (6–4) dimers. Once activated by light, photolyases are able to catalyse the reverse of some of these damaging reactions, restoring the original DNA structure (Sancar 2016). The chemical process is initiated by reduction of fully oxidised FAD to FAD·^−^ or FADH· and finally through to FADH^−^, with the reduced species providing electrons to cleave bonds in the defective DNA (Müller et al. 2015). Alongside this is an antenna chromophore with a different absorption range that broadens spectral properties. Five of these antenna chromophores have been identified so far for photolyase/cryptochrome superfamily members (Kiontke et al. 2014). Alternatively, but based on the same chemistry, cryptochromes are core components of circadian rhythms, which allow organisms, including humans, to respond behaviourally to the daily cycle of light and dark, by initiating and shutting down biochemical processes as appropriate (Mei and Dvornyk 2015). Cryptochromes are also proposed to have a role in magnetoreception (Hore and Mouritsen 2016). Importantly for our work, the efficiency of the radical-pair mechanism appears to depend on precise details of the amino acid sequence of the cryptochrome in a given species, in particular a “dyad”, “triad” or “tetrad” of tryptophan residues (Müller et al. 2015; Cailliez et al. 2016). We aimed to carry out a wide-ranging phylogenetic analysis of the photolyase/cryptochrome superfamily, building on earlier work (see below), and to map the presence of tryptophan motifs across the resulting phylogenetic tree.

The aim of this study was to ascertain the bacterial contribution to the function and evolution of the photolyase/cryptochrome superfamily and hence inform models of eukaryogenesis. Previous phylogenetic analyses have taken place within this family (Kanai et al. 1997; Öztürk et al. 2007; Mei and Dvornyk 2015; Scheerer et al. 2015). The most recent comprehensive phylogenetic analysis of the photolyase/cryptochrome superfamily in eukaryotes was published by Mei and Dvornyk in 2015 (Mei and Dvornyk 2015), who noted the existence of prokaryotic orthologues but included only a few bacterial sequences in their analysis. Earlier, Lucas-Lledó and Lynch had undertaken another major phylogenetic analysis including numerous prokaryotic photolyase/cryptochromes (Lucas-Lledó and Lynch 2009). However, their study focused mainly on photolyase gene loss, whereas we are interested in the *acquisition* of particular biochemical features. Also, very many prokaryotic genomes have been sequenced since 2009. Therefore, we decided to start from the eukaryotic analysis from Mei and Dvornyk, to carry out our own search for prokaryotic orthologues and to cross-reference our findings to the work of Lucas-Lledó and Lynch. Previously it had been suggested that both gene duplication events, gene loss and convergent evolution may have taken place across this family of proteins, in particular during the evolution of cryptochromes from plants and animals, known as Plant Cry and Animal Cry, respectively (Cashmore et al. 1999).

## Results

### Phylogenetic Analysis of Currently Available Prokaryotic and Eukaryotic Genomes Supports the Existence of Seven Subfamilies of Photolyase/Cryptochromes

**Fig. 1:**
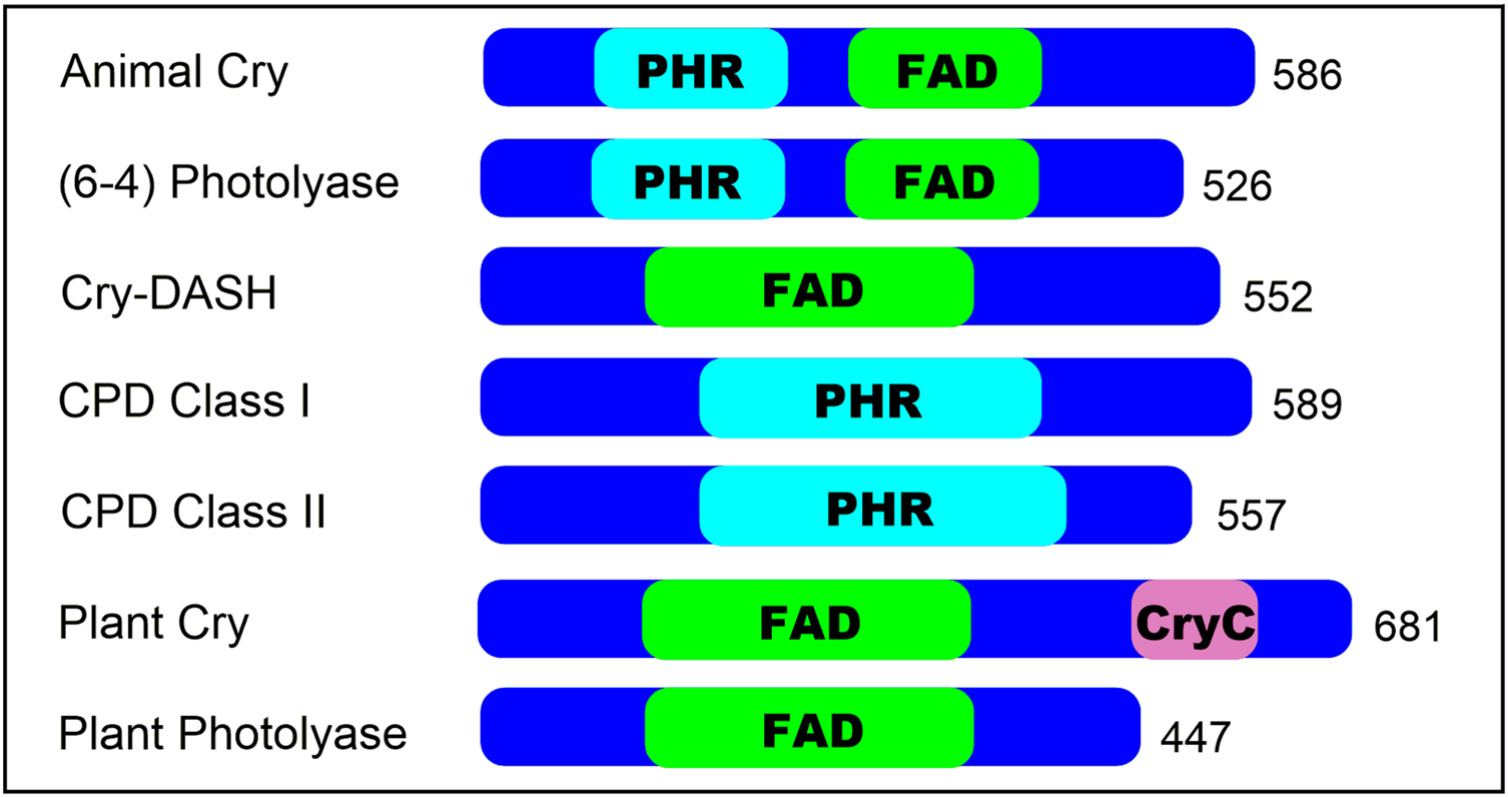
The seven subfamilies used in phylogenetic analysis. Main representatives are shown alongside their predicted domains based on NCBI. *PHR* photolyase, *FAD* FAD-binding domain, *CryC* Cryptochrome C

To start our search for candidate bacterial orthologues, we selected seven of the sequences used by Mei and Dvornyk (2015) as queries for their analysis. Listed in the Materials and Methods, these cover the previously identified subfamilies (6–4) Photolyase/Animal Cry, Plant Cry, Plant Photolyase, Cyclobutane Pyrimidine Dimer (CPD) Class I Photolyase, CPD Class II Photolyase and Cry-DASH (Fig. 1). The top 20 candidate orthologues from different bacterial species were selected from protein–protein BLAST (BLASTp) searches of the NCBI non-redundant protein database, filtered for taxid:2 (bacteria). We found no candidate bacterial orthologues of Plant Photolyase proteins using this method. In order to confirm that the identified sequences were candidate orthologues, these were then used in a reciprocal BLASTp search against the original species to see if the starting sequence was obtained as the best match. Sequences with complete genomes were kept and sequences obtained from metagenomes were removed when the genome completion was below 50%. Extra eukaryotic and archaeal sequences, beyond those used as start points for our BLASTp searches, were added to give a more complete picture of the photolyase/cryptochrome superfamily including species from most major taxa. We accrued 186 amino acid sequences in our database (full details in Supplementary Information).

According to eggNOG, 84% of orthologues of “cryptochrome/DNA photolyase, FAD-binding domain” are found in bacteria, 14% in eukaryota and 2% in archaea. The bacterial total of 84% comprises 45% from proteobacteria (15% *α*-, 6% *β*- and 22% *γ*-proteobacteria), 16% from actinobacteria, 8% from bacteroidetes/chlorobi, 5% from cyanobacteria, 5% from firmicutes and 5% other. A similar spread across the bacterial phyla is observed in OrthoDB. We have ensured there are representatives of all these phyla in our database.

We aligned our protein sequences using MUSCLE (Multiple Sequence Comparison by Log-Expectation) (Edgar 2004) to directly compare it to the analysis undertaken by Mei and Dvornyk. To reinforce the reliability we also aligned our chosen sequences using MAFFT (Multiple Alignment using Fast Fourier Transform) (Katoh et al. 2017) and PRANK (Löytynoja and Goldman 2010) for further comparison. Following alignment and trimming of sequences using Gblocks (Castresana 2000), trees were generated using PhyML with approximate likelihood ratio test and Shimodaira-Hasegawa (aLRT-SH) used as an estimate for branch support in PhyML and the posterior probabilities shown for the Bayesian analysis. Again these are the same methods used by Mei and Dvornyk, with the exception that their alignment was trimmed manually using Bioedit, where we used Gblocks (Mei and Dvornyk 2015). The low stringency setting selected in Gblocks ensured that only isolated elements of particular sequences were removed. The LG (Le and Gascuel) model was used in all cases for tree generation, as chosen by smart model selection through the PhyML software (Le and Gascuel 2008).

The three resulting trees are presented in Figures S1 (MUSCLE), S2 (MAFFT) and S3 (PRANK). The previous analysis (Mei and Dvornyk 2015) aligned the sequences using MUSCLE. Hence we present the analysis based on MUSCLE here as our primary output. Our trees are presented unrooted, as is the case for trees from Lucas-Lledó and Lynch (2009) and Mei and Dvornyk (2015). The latter work devoted significant effort to establishing the geological timeline of the evolution of the photolyase/cryptochrome superfamily, giving confidence that CPD Class II Photolyases are basal to the superfamily. From our biochemical perspective, it is sensible to effectively use the CPD Class II Photolyases as an outgroup for the analysis, since, as discussed below, they only possess a “dyad” of tryptophan residues as compared with the triads and tetrads that are characteristic of the other subfamilies.

**Fig. 2:**
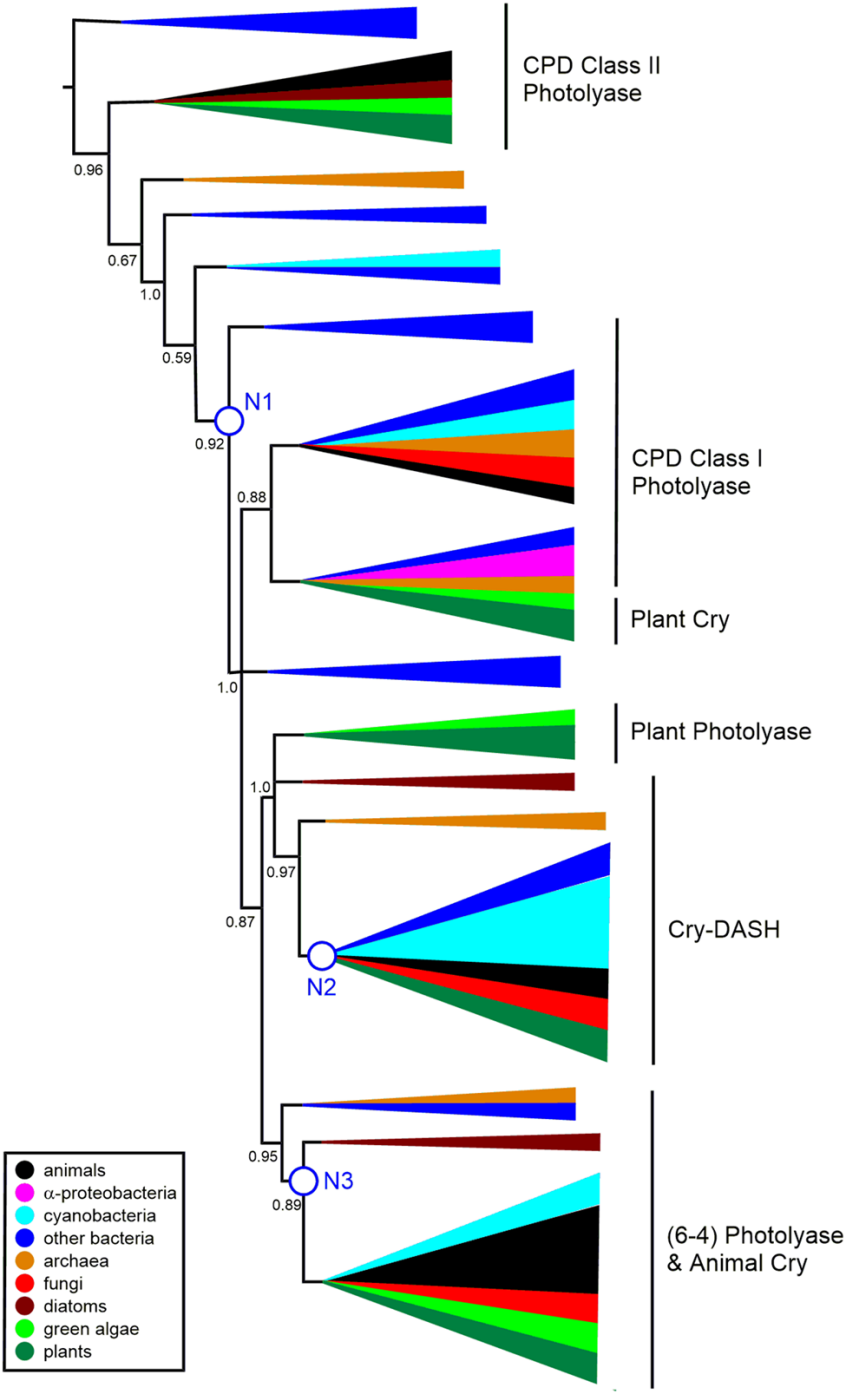
Schematic phylogenetic tree from MUSCLE alignment. The full tree is in Supplementary Figure S1. aLRT-SH over 0.5 are shown, with branches below 0.5 being collapsed. We have not attempted to precisely define the different clades, but have indicated the locations of the main subfamilies with reference to Fig. 1 and Mei and Dvornyk (2015). N1–N3 refer to the three nodes discussed in the manuscript. The Figure highlights the positions of animal sequences (in black) relative to α-proteobacterial sequences (in magenta) and cyanobacterial sequences (in cyan). Also colour coded are sequences from other bacteria (blue), archaea (orange), fungi (red), diatoms (brown), green algae (light green) and plants (dark green) (Color figure online)

The (6–4) Photolyase/Animal Cry subfamily, the Cry-DASH subfamily and the Plant Photolyase subfamily appear in our Figures S1 and summary Fig. 2 much as they do in the equivalent tree from Mei and Dvornyk. The relative positions of the CPD Class I Photolyase and Plant Cry subfamilies show less consistency between our tree in Fig. 2 and Mei and Dvornyk’s results. However, the tree based on our MAFFT alignment (Fig. S2) exhibits more similarity. We should also note that the literature phylogenetic tree that includes more bacterial sequences (Lucas-Lledó and Lynch 2009) places the CPD Class I Photolyase and Plant Cry subfamilies quite differently. We conclude that our findings both broadly accord with the literature and reflect the unresolved subtleties of the previous analyses.

We should note an interesting set of orthologues from *Chloroflexi* bacteria. These five proteins form a monophyletic group in all three of our trees (Figs. S1–S3), but are located differently in each case with respect to the six subfamilies. When these sequences are modelled they appear to be most similar to CPD Class III Photolyases (these sequences align best with 4U63 from the CPD Class III *Agrobacterium tumefaciens* structure with 42% sequence identity between *Roseiflexus* orthologue and 43.38% homology with *Chlorflexus aggregans* orthologue) (Scheerer et al. 2015).

### Incongruences in the Evolution of the Superfamily

The occurrence of horizontal gene transfer from ancestral bacteria to ancestral eukaryotes is widely accepted to have resulted from the endosymbioses that formed mitochondria and also, in the case of plants, chloroplasts. More recently, further examples of possible HGT have started to emerge. Indeed Lucas-Lledó and Lynch have mentioned the importance of gene gain, by HGT, as well as gene loss during the evolution of the photolyase/cryptochrome superfamily (Lucas-Lledó and Lynch 2009). However, these authors did not explore in detail how such HGT events contribute to the acquisition of new function during evolution and this is our primary focus.

To identify putative HGT occurrences, we have followed guidance from authors including Husnik and McCutcheon (2018) who conclude that "Phylogenetic conflict (that is, incongruence of a single-gene tree with a known species phylogeny) is the method of choice for the detection of HGT events.” We have identified three significant incongruences in our photolyase/cryptochrome “single-gene tree” that are visible in all three Figures S1–S3. The relevant nodes are annotated in summary Fig. 2 as N1, N2 and N3. Node 1 (N1) represents a common ancestor of photolyases and cryptochromes from various taxa of bacteria, from archaea, from algae, fungi, plants and animals. However, this appears more recently than the common ancestor of animal CPD Class II Photolyase proteins and the other animal paralogues in the superfamily. This is clearly incongruent with the standard tree of life. Node 2 (N2) represents the most recent common ancestor of Cry-DASH proteins from a group of eukaryotes, including animals, and of bacteria, including numerous cyanobacterial proteins. Again, the standard model would not predict this to fall more recently than the common ancestor of the eukaryotic members of the superfamily. Node 3 (N3) represents the most recent common ancestor of cryptochromes (Cry) from animals, fungi, plants, algae and, remarkably, two species of *Gloeobacter*, a further striking incongruence. This last feature was noted by Lucas-Lledó and Lynch (2009), though *G. violaceus* was the only *Gloeobacter* genome then available. They invoked a HGT from green algae to *G. violaceus*.

The trees from Lucas-Lledó and Lynch (2009) and Mei and Dvornyk (2015) show a node, similar to our Node 1, that represents a common ancestor of all cryptochrome/photolyase proteins, except CPD Class II Photolyases, in most major phyla of bacteria and eukaryotes. They also both show a node, similar to our Node 2, that represents a common ancestor of (6–4) Photolyase/Animal Cry and bacterial Cry-DASH sequences (though Lucas-Lledó and Lynch did not label this group as Cry-DASH). Our tree is the first to include sequences from both sequenced species of *Gloeobacter*, but the location of Node 3 accords with the placement of *G. violaceus* in the literature trees. These previous authors did not discuss these apparent incongruences in detail.

We should also note the presence of archaeal sequences in our data set. Apart from one sequence from *Thaumarchaeota*, all the archaeal species represented are *Euryarchaeota*, in which HGT from bacteria is a common feature (see for example Santa-Molina et al. 2020). Therefore, the presence of photolyase/cryptochrome superfamily genes in these archaea is not informative with respect to the analysis presented here.

### Conserved Residues and Hence Functionalities are Observed Across All Families

**Fig. 3:**
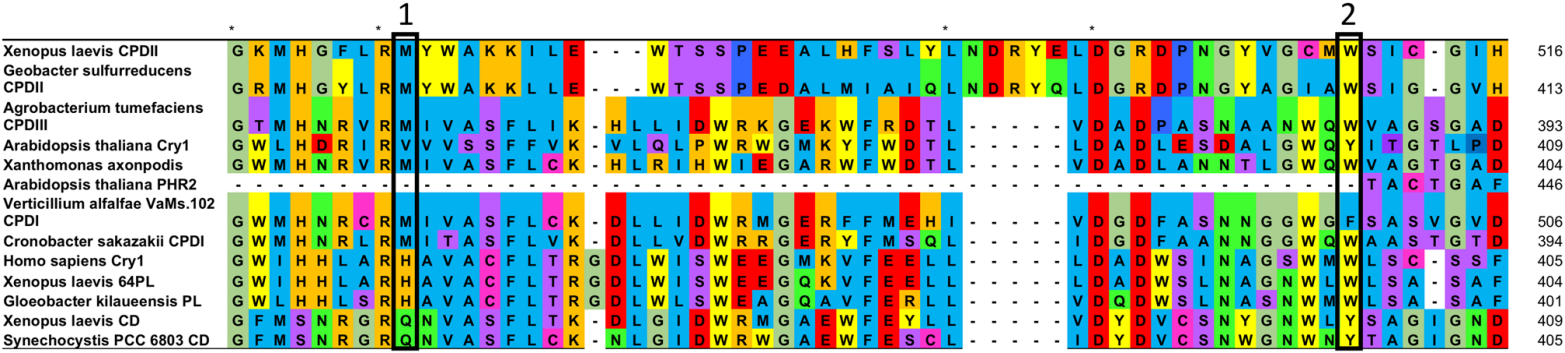
Alignment of examples of each subfamily alongside bacterial orthologues for each (aside from CPD III which is characterised from the bacteria *A. tumefaciens* and from Plant Photolyase *A. thaliana* which has no bacterial orthologues). Residues involved in substrate recognition are highlighted with a black box and whole conserved residues are shown with an *. Sequences used are *X. laevis* (6–4) PL (NP_001081421.1), *X. laevis* CPD II (NP_001089127.1), *X. laevis* CD (NP_001084438.1), *Geobacter sulfurreducens* CPD II (WP_010943456.1), *Agrobacterium tumificans* (WP_010971478.1), *A. thaliana* Cry1 (NP_567341.1), *A. thaliana* PHR2 (NP_182281.1), *X. axonpodis* (WP_011050942.1), *V. alfalfae* (XP_002999933.1), *Cronobacter sakazakii* WP_012125345.1, *H. sapiens* Cry1 (NP_004066.1), *G. kilaueensis* (WP_023172734.1), *Synechocystis* PCC 6803 CD (WP_014407097.1) (Color figure online)

Previously Zhang et al (2017) identified conserved residues in the superfamily that relate to DNA binding within the photolyase, defining which substrate is recognised and repaired by the photolyase (Table 1 and Fig. 3). In Fig. 3 they are labelled as position 1 and position 2. Looking at position 1 we can see that, like their eukaryotic counterparts, bacterial orthologues of CPD Photolyase proteins have a methionine in this position. The cyanobacterial *Gloeobacter* sequences that surprisingly group with (6–4) Photolyase/Animal Cry have the characteristic histidine at the same position and bacterial orthologues of Cry-DASH have the expected glutamine.

**Table 1:** Conserved residues identified across members of the superfamily

Subfamily	Position 1	Position 2	Substrate
(6–4) photolyase	H	W	6–4
Animal Cry	H	W	–
CPD class I	M	W	CPD
CPD class II	M	W	CPD
CPD class III	M	W	CPD
Cry-DASH	Q	Y	ssDNA
Plant Cry	V	Y	–
Plant photolyase	–	–

Conversely, in position 2, all of the members of the CPD Photolyase subfamilies have a tryptophan at this position apart from the fungal CPD Photolyase, which, in comparison to its bacterial orthologue, contains a tyrosine. The bacterial orthologues of Cry-DASH retain the tyrosine also at position 2. The *Gloeobacter* sequences again have the conserved tryptophan seen in the rest of the (6–4) Photolyase/Animal Cry subfamily. The bacterium *Xanthomonas axonpodis* that groups with the Plant Cry subfamily appears to have residues more like a CPD Photolyase at these positions (M358 and W397). The Plant Photolyase member doesn’t convey any conserved residues at these positions. Conservation of these residues in the bacterial sequences in our study gives confidence that the orthologues are genuine and that they will have the same function in vivo as eukaryotic members of the same subfamily.

### The “Canonical” Tryptophan Triad and Tryptophan Tetrad Motifs have Emerged Since the Divergence with CPD Class II Photolyases

**Fig. 4:**
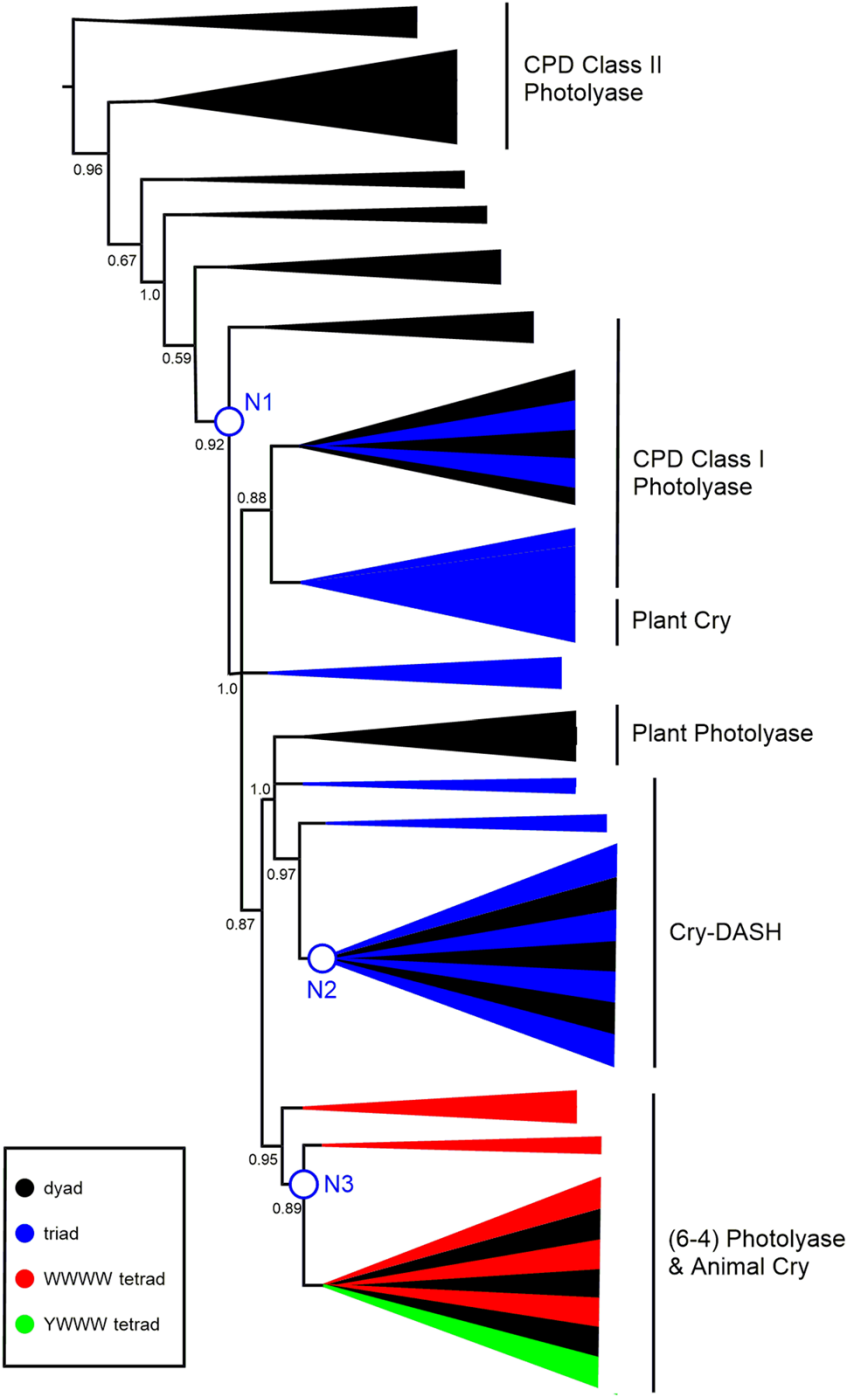
Schematic phylogenetic tree from MUSCLE alignment. aLRT-SH over 0.5 are shown, with branches below 0.5 being collapsed. N1–N3 refer to the three nodes discussed in the manuscript. Proteins that contain the “canonical” tryptophan triad are represented by blue branches. Proteins with a WWWW tetrad are represented by red branches and with a YWWW tetrad green. The canonical triad is found widely through the CPD Class I, Plant Cry and Cry-DASH subfamilies. Tetrads are found mainly in the (6–4) Photolyase/Animal Cry subfamily. The full tree is in Supplementary Figure S4 (Color figure online)

Crucial to the function of some members of this superfamily is a triad of tryptophan residues that are required for the transmission of the electron from FAD to create FADH which is then, for example, used for the repair of DNA in (6–4) Photolyases (Hitomi et al. 2009). We mapped the presence of these three tryptophans on our phylogenetic tree and observe that this mechanism appears to have evolved more recently than the divergence from CPD Class II Photolyase proteins (Fig. 4). The three tryptophan residues are present in the majority of orthologues that have evolved since Node 1. In subsequent research, the tryptophan triad that is involved in electron transfer to FAD has been expanded to include a further tryptophan in *Drosophila* and *Xenopus* (Müller et al. 2015; Nohr et al. 2016; Yamamoto et al. 2017; Lin et al. 2018)**.** This tryptophan “tetrad” increases the lifetime of FADH. The fourth tryptophan in the tetrad is replaced by a tyrosine in algae and plants, which is able to produce a similar effect (Nohr et al. 2016; Oldemeyer et al. 2016; Franz et al. 2018)**.**

**Fig. 5:**
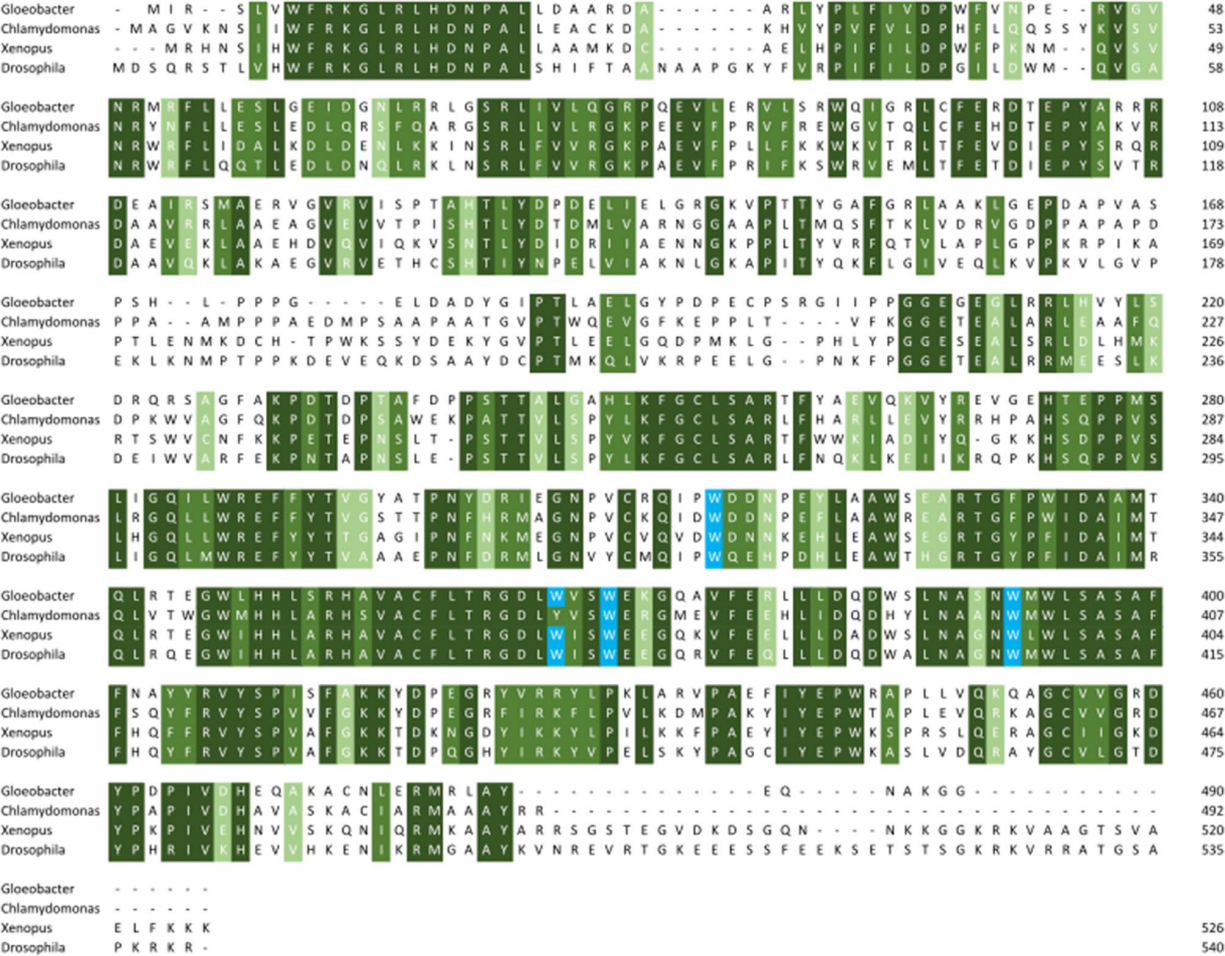
Alignment of (6–4) Photolyase orthologues*. Gloeobacter violaceus* (6–4) PL **(**WP_011141747.1)*, X. laevis* (6–4) PL (NP_001081421.1), *Chlamydomonas reinhardtii* (6–4) PL (XP_001698054.1) and *Drosophila melanogaster* (6–4) PL (BAA12067.1). Residues in the tryptophan tetrad are highlighted in blue, with the degree of conservation between sites shown in green, with darkest green being entirely conserved at that position. *Gloeobacter* is 50% identical to the amphibian *Xenopus*, 50% to the insect *Drosophila*, 56% to the alga *Chlamydomonas* (Color figure online)

**Fig. 6:**
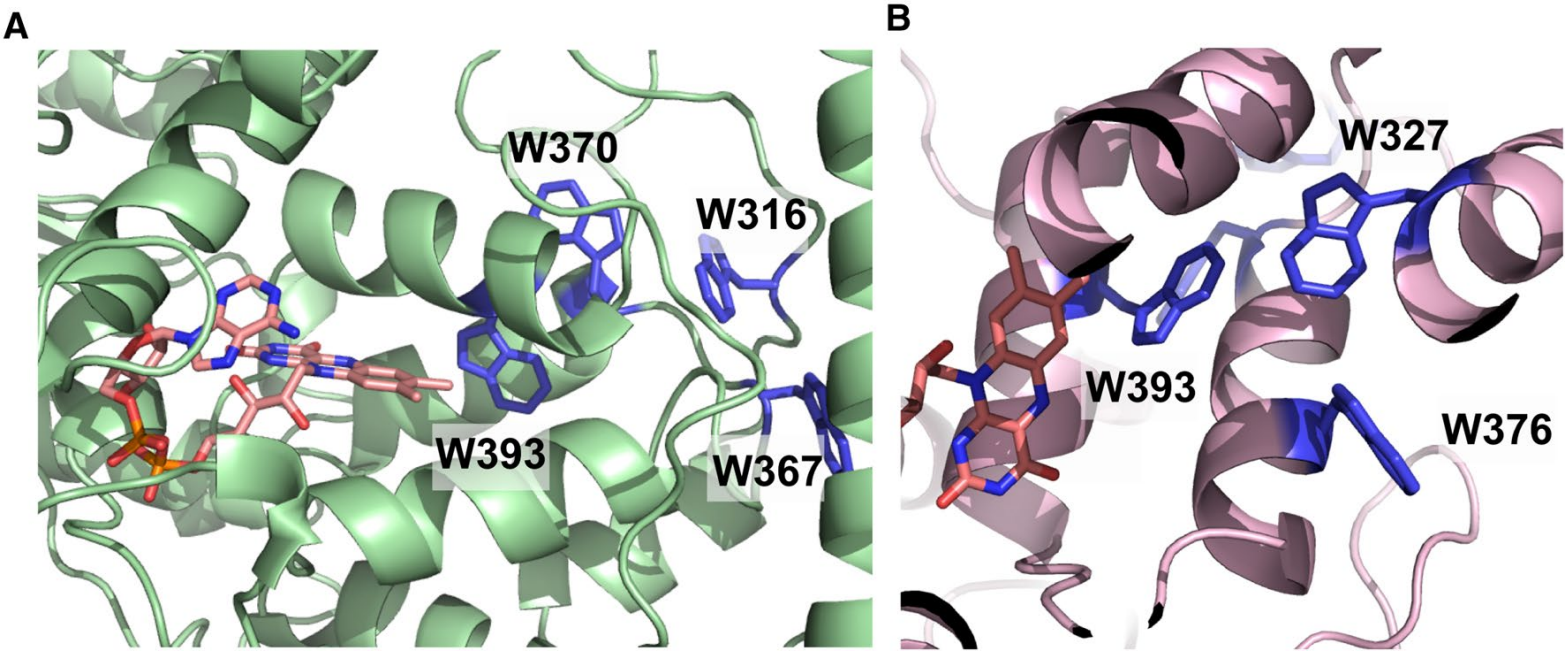
**a** Model of (6–4) Photolyase orthologue from *G. kilaueensis* using Phyre 2. The conserved tryptophan tetrad is shown in blue with FAD modelled in from 3FY4 shown in peach. **b** Model of a Cry-DASH orthologue from *G. kilaueensis* with the “alternative” tryptophan triad shown in blue (Color figure online)

We wondered when this tryptophan tetrad motif evolved, so looked for the presence of the fourth tryptophan throughout our phylogenetic analysis. The sequences that contain this fourth tryptophan are shown on the phylogenetic tree in Fig. 4 with red branches. All of the animal cryptochromes have this fourth tryptophan, usually in a four amino acid motif of *W*(*Φ*)SW, where the first Trp is the newly identified member of the “tetrad”. We also identified this motif in the two sequenced *Gloeobacter*, which are cyanobacteria (*Gloeobacter kilaueensis* WP_023172734.1 and *Gloeobacter violaceus* WP_011141747.1) (Figs. 5 and 6). These proteins group near the algal (6–4) Photolyases, which are able to undertake DNA repair and gene control using an unusual arrangement of long C-terminal extension (Kottke et al. 2017; Franz et al. 2018). As mentioned above, the algal proteins mainly have a *Y*(*Φ*)SW motif instead of *W*(*Φ*)SW and hence a YWWW version of the WWWW tetrad.

We should note that CPD Class II Photolyases use a different set of tryptophans from the rest of the classes. This tryptophan ‘dyad’ was defined by Kiontke et al. (2011). All of the CPD Class II Photolyase orthologues in this analysis contain these two tryptophans (numbered W449 and W470 in *Xenopus laevis* CPD II). These proteins also contain a conserved tyrosine, suggesting that the electron transfer in this subfamily differs from the usual all tryptophan mechanism (Y434 in *X. laevis* CPD II). As mentioned earlier, this provides a firm biochemical justification for treating CPD Class II Photolyases as, essentially, an outgroup in our phylogenetic analysis.

### An “Alternative” Tryptophan Triad is Characteristic of Cry-DASH Proteins

Whilst the canonical triad (and tetrad) of tryptophan residues is characteristic of all subfamilies of the photolyase/cryptochrome superfamily except CPD Class II Photolyases, experiments in *Escherichia coli* and *Arabidopsis thaliana* have shown that this particular triad is not always essential for function (Y. F. Li et al. 1991; X. Li et al. 2011). Indeed, Biskup et al*.* (2011) showed in the cyanobacterium *Synechocystis* that an “alternative” triad of tryptophan residues could mediate electron transfer (Fig. 6b). Two of the canonical tryptophan residues still contribute to the alternative set, but in *Synechocystis* a W320 completes the triad, not W375. In some orthologues, the redundant tryptophan is lost.

**Fig. 7:**
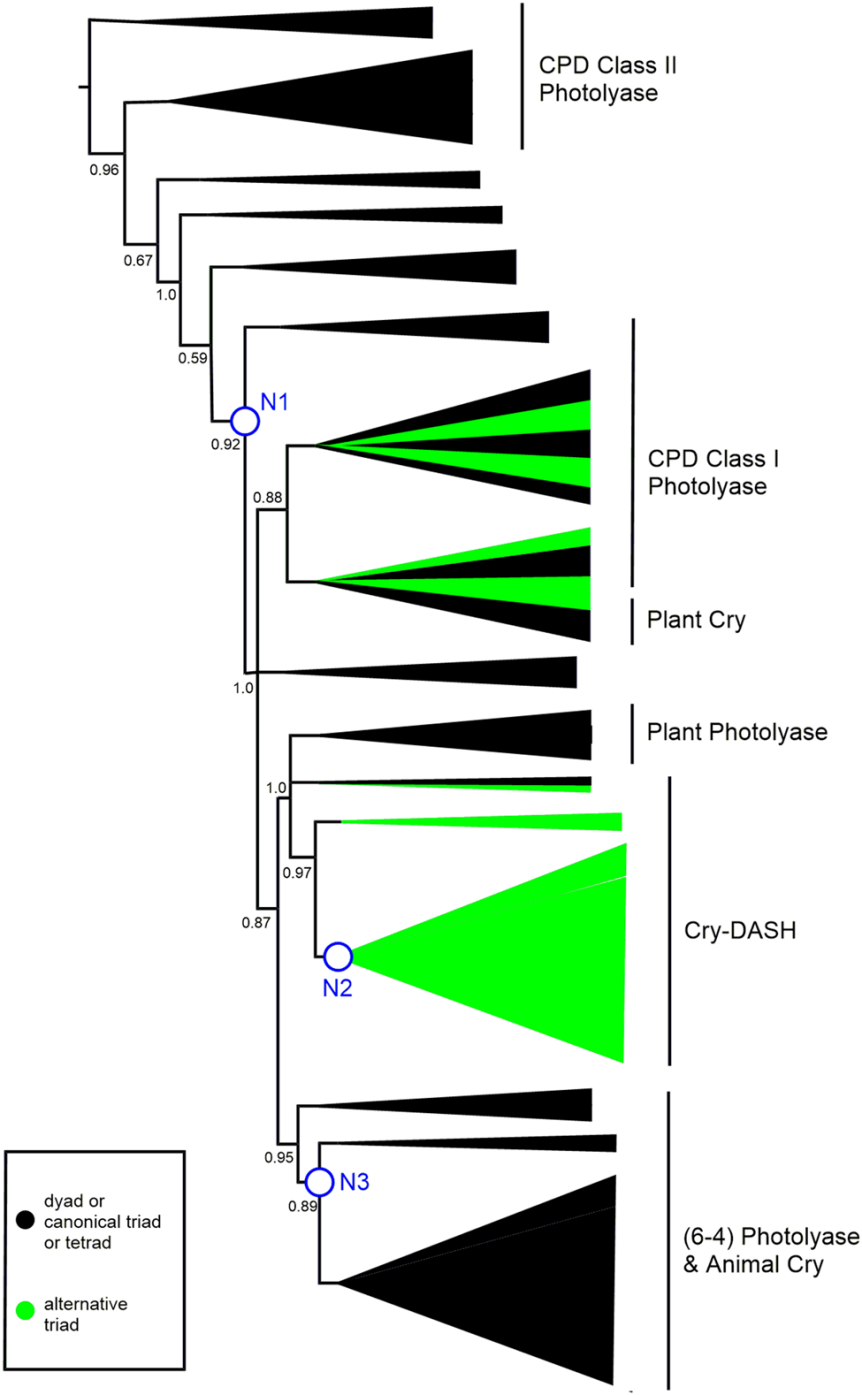
Schematic phylogenetic tree from MUSCLE alignment. aLRT-SH over 0.5 are shown, with branches below 0.5 being collapsed. N1–N3 refer to the three nodes discussed in the manuscript. Clades with proteins that exhibit the “alternative” tryptophan triad are highlighted either in green, where almost all examples have the alternative triad (Cry-DASH), or green and black where some but not all examples have the alternative triad, as in the CPD Class I Photolyase and Plant Cry subfamilies. The full tree is in Supplementary Figure S5 (Color figure online)

In Figures S5 and S7, sequences containing the “alternative” triad of tryptophan residues are indicated with green coloured branches (Fig. 7). Importantly, the identification of the different triads in our work rests solely on the alignment of the sequences. In many cases, the proteins possess the elements of both the canonical and alternative triads and, with the exception of *Synechocystis* (see above), we do not know which of them is functional. The alternative triad is found sporadically amongst CPD Class I Photolyases, including those that use a different cofactor (5,10-methylenetetrahydrofolate, MTHF) and that are often called CPD Class III Photolyases (Scheerer et al. 2015). The alternative pattern is not found at all in Plant Photolyase proteins, nor in (6–4) Photolyase/Animal Cry.

Strikingly, the alternative tryptophan triad appears to be an essential feature of Cry-DASH proteins, being present in all the Cry-DASH sequences in our phylogenetic tree, with the exception of the proteins from diatoms *Thalassiosira pseudodonna* and *Phaeodactylum tricornutum* (Figure S5). In fact, the *P. tricornutum* protein has been shown experimentally to more closely resemble a Plant Photolyase in function, hence the annotation as Cry-DASH could be revisited (Juhas et al. 2014).

## Discussion

We present here a phylogenetic analysis of cryptochrome and photolyase proteins that includes an array of prokaryotic sequences. The tree in Fig. 2 has all the hallmarks of the previous definitive analysis of this superfamily from Mei and Dvornyk. However, similar to our previous research on retinoic acid biosynthesis (Miles et al. 2019; Millard et al. 2014), we find that inclusion of photolyase/cryptochrome sequences and candidate orthologues from across the kingdoms of life brings to the fore phylogenetic relationships that are particularly relevant to eukaryogenesis, in particular with respect to the placement of animal and bacterial orthologues.

We have highlighted three striking incongruences between our gene tree for the photolyase/cryptochrome superfamily and the widely accepted species tree that is based on analysis of ribosomal protein sequences (Hug et al. 2016). The simplest hypothesis that accords with this observation is to propose at least three HGT events between ancestral bacteria and ancestors of eukaryotes that have contributed to the emergence of this superfamily of proteins.

Mapping the occurrence of tryptophan dyads, canonical and alternative triads and tetrads across the phylogenetic tree is revealing. Broadly, we can say that CPD class II photolyase proteins possess a dyad and are thus predicted to be the least effective at stabilising diradicals. Indeed, we are essentially using the CPD Class II Photolyase sequences as an outgroup in this study. We then see acquisition of triad motifs that approximately coincides with the putative HGT at Node 1. Strikingly, within the Cry-DASH subfamily that has a common ancestor at Node 2, we find that the “alternative” triad is completely conserved, again indicating this feature may have accompanied HGT. Finally, around Node 3, where we propose a further HGT, we see appearance of tryptophan tetrad motifs. These tryptophan signatures provide powerful additional support for multiple HGT events.

In Fig. 2, we highlight where candidate bacterial orthologues from two key groups of bacteria, namely *α*-proteobacteria and cyanobacteria, are located, to clarify how the incidences of HGT we propose relate to the widely accepted endosymbiotic gene transfer (EGT) events, that is EGT between an ancestral *α*-proteobacterium and an ancestor of all eukaryotes and EGT between an ancestral cyanobacterium and an ancestor of plants. From an animal perspective, if we ignore isolated branches for sequences from each of *Tetrahymena thermophila* and *Trypanosoma brucei*, we find animal photolyase/cryptochrome superfamily proteins in only three clades, namely CPD Class II Photolyase, Cry-DASH and (6–4) Photolyase/Animal Cry. If EGT is the source of bacterial genes in animals, then we would expect to observe animal sequences having more recent common ancestors with *α*-proteobacterial proteins than with other bacterial groups in these CPD Class II Photolyase, Cry-DASH and (6–4) Photolyase/Animal Cry clades.

In fact, in our tree, *α*-proteobacterial sequences are only found in the Plant Cry clade. Lucas-Lledó and Lynch did identify some *α*-proteobacterial orthologues of Cry-DASH in their study, but they were not recovered by our methods. This is easy to understand from a simple BLASTp of Cry-DASH from, for example, *X. laevis*. There are hundreds of hits from cyanobacteria, firmicutes, CFB group bacteria etc*.* that score more highly than the closest *α*-proteobacterial match.

So, if we focus on animals, *α*-proteobacteria and cyanobacteria, we find that animal (6–4) Photolyase/Animal Cry genes have a more recent common ancestor with cyanobacteria (specifically two *Gloeobacter* species) than with *α*-proteobacteria (see Node 3) and we find that animal Cry-DASH genes also have a more recent common ancestor with cyanobacteria than with *α*-proteobacteria (see Node 2). Gabaldón (2018) and López-García and Moreira (2020) argue that is very difficult to reconcile such observations with just an EGT from an *α*-proteobacterium to form the mitochondrion. We would need to assume that orthologues of Animal Cry and of animal Cry-DASH genes had been lost in all sequenced extant species of *α*-proteobacterium (with the exception of those mentioned above where there are “distant” orthologues). Rather, evidence is accumulating for “waves” of acquisition of genes from different bacterial sources in addition to the endosymbiosis to form the mitochondrion and Gabaldón (2018) says “we need to go beyond simple models that involve a single bacterial endosymbiont engulfed by an archaeal ancestor”.

**Fig. 8:**
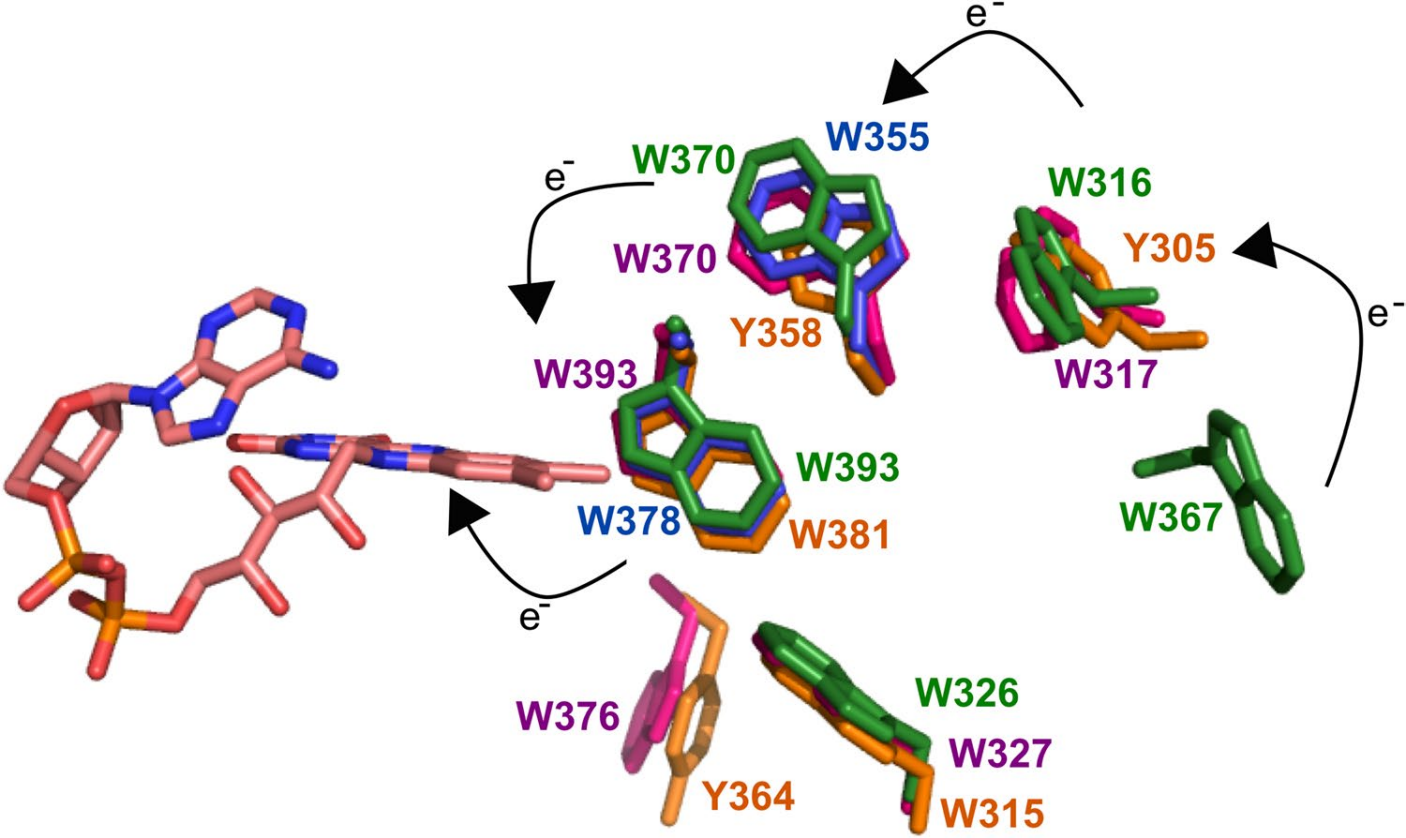
All four photolyase/cryptochrome family members from *G. kilaueensis* modelled with conserved tryptophans shown. Green—(6–4) Photolyase that contains the fourth tryptophan, Blue—CPD Class II like orthologue that comprises a tryptophan dyad, Pink—Cry-DASH. Orange—unannotated paralogue. All structures were modelled using Phyre 2.0 (Color figure online)

Intriguingly, the two *Gloeobacter* species in our analysis have photolyase/cryptochrome proteins that exhibit all of the canonical triad, the alternative triad and the tetrad of tryptophan residues (Fig. 8). The *Gloeobacter* proteins with the tryptophan tetrad are annotated as (6–4) Photolyases in both *G. kilauensis* and *G. violaceus* and the proteins with the characteristic alternative triad are annotated, consistent with our observation on the Cry-DASH group above, as Cry-DASH in both species. A further pair of *Gloeobacter* proteins group with a number of CPD Class I Photolyases that are believed to use 8-HDF as the second cofactor. Finally, *G. kilauensis* has yet another, unannotated, paralogue.

*Gloeobacter* species are generally accepted to have branched from the rest of the cyanobacteria very early in evolutionary history (Schirrmeister et al. 2013). *Gloeobacter* species lack thylakoid membranes that are the normal sites in cyanobacteria of light-dependent reactions. *G. kilaueensis* was discovered in a Hawaiian lava cave in 2013 (Saw et al. 2013), whilst *G. violaceus PCC 7421* was isolated from a limestone rock surface (Nakamura et al. 2003; Mareš et al. 2013). To confirm that the seven photolyase/cryptochromes all most probably evolved as paralogues in *Gloeobacter*, the GC contents of the genes were compared with that of the overall organism. All seven of the photolyase/cryptochrome genes in *Gloeobacter* have GC contents that are consistent with that of the whole organism (Table 2), which is evidence against acquisition of these genes by a more recent HGT to the cyanobacteria, as proposed by Lucas-Lledó and Lynch (2009).

**Table 2:** The GC content of orthologues identified in Gloeobacter compared to the GC content of the complete genome

Organism	Annotation in phylogenetic analysis	Genome content	Gene content	Accession code	Closest orthologue
*Gloeobacter kilaueensis* JS1	Gloeobacter_kilaueensis	60.5	60.89	WP_023175030.1	CPD Class III
	Gloeobacter_kilaueensis_DASH		62.97	WP_023173156.1	Cry-DASH
	Gloeobacter_kilaueensis_PL		62.74	WP_023172734.1	(6–4) Photolyase
	Gloeobacter_kilaueensis_PL2		63.45	WP_023172026.1	CPD
*Gloeobacter violaceus* PCC 7412	Gloeobacter_violaceus	62	62.3	WP_011141747.1	(6–4) Photolyase
	Gloeobacter_violaceus_CPD		64.64	WP_011140786.1	CPD
	Gloeobacter_violaceus_DASH		60.733	WP_011140837.1	Cry-DASH

Overall, our analysis suggests that bacteria have contributed significantly *and repeatedly* to the evolution of photolyases and cryptochromes across all kingdoms of life, including in animals, by putative HGT. These apparent gene transfer events seem to be linked to the acquisition of additional and/or different tryptophan residues that contribute to increased stability of the diradical intermediate that is so crucial to the mechanism of photoreception. We are mindful of the dangers of inferring features of species trees from single gene trees (Ku et al. 2015) and that is not our intention. Rather we aim to show that incongruent nodes in this gene tree align with fundamental distinctions in the biochemistry of different groups of photolyase/cryptochrome proteins, which invites further investigation. Indeed, case studies such as the complex phylogeny of the photolyase/cryptochrome superfamily could contribute valuable insight to emerging models of eukaryogenesis that acknowledge a rich bacterial heritage.

## Materials and Methods

### Phylogenetic Analyses

Cryptochrome and photolyase starting sequences were chosen from (Mei and Dvornyk 2015) and BLASTp searched against the NCBI non-redundant protein database against bacteria with the default settings to identify orthologous proteins. The initial sequences were Animal CRY (*Homo sapiens* NP_004066 and NP_066940), (6–4) Photolyase (*X. laevis* NP_001081421), CPD Class II Photolyase (*Monosiga brevicollis* MX1 XP_001746666), Cry-DASH (*Salpingoeca *sp. ATCC 50,818 XP_004989008), Fungal CPD Class I Photolyase (*Verticillium alfalfae* VaMs.102 XP_002999933); Plant Cry (*A. thaliana* NP_567341 and NP_171935); Plant Photolyase (*A. thaliana* NP_182281). The top 20 bacterial orthologues for all subfamilies were chosen, along with orthologues from all other groups of life. Full details of sequences collected are in the Supplementary Information. Some sequences were removed when they were found to be from incomplete genome sequences such as those derived from metagenomes.

Amino acid sequences were aligned using MAFFT with method FFT-NS-I (Katoh et al. 2017), MUSCLE (Edgar 2004) and PRANK (Löytynoja and Goldman 2010). Redundant regions of the alignment were then removed using Gblocks with low stringency settings (Minimum number of seq for a conserved and flank position set to half, Maximum number of contiguous nonconserved positions: 50, Minimum length of a block: 2, All gap positions allowed) (Castresana 2000). This retained 739 positions out of 2049 with the MUSCLE alignment (36% in 5 blocks), 732 positions out of 2075 with the MAFFT alignment (38% in 6 blocks) and 697 positions out of 5842 in the PRANK alignment (12% in 10 blocks). Phylogenetic trees were then built using PhyML with approximate likelihood ratio testing giving branch support values and the Le and Gascuel (LG) model used which had been chosen in Smart Model Selection (Le and Gascuel 2008; Guindon et al. 2010; Lefort et al. 2017). Trees were analysed and annotated using Treegraph2 (Stöver and Müller 2010).

### GC Content Calculation

Genomes in the NCBI were used to identify the GC content of the entire genome of a bacteria and compare this to the GC content of the gene. A disparency of 8% was the cutoff set for a gene to be ‘real’ and not a possible contaminant.

### Modelling Cyanobacterial Proteins

The structure of *G. kilaueensis* (WP_023172734.1**)** and *G. violaceus* (WP_011141747.1) (6–4) PL were modelled using Phyre 2.0 (Kelley et al. 2015) with 3FY4 as the template (Hitomi et al. 2009). The Cry-DASH orthologues were modelled with 1NP7 as the template (*G. kilaueensis* WP_023173156.1 and *G. violaceus* WP_011140837.1) (Brudler et al. 2003). The 8-HDF like orthologues were modelled with 1TEZ as the template (*G. kilaueensis* WP_023172026.1 and *G. violaceus* WP_011140786.1) (Mees et al. 2004). Finally, the other *G. kilaueensis* orthologue was modelled with 4U63 as a template (WP_023175030.1) (Scheerer et al. 2015).

## Electronic supplementary material

Below is the link to the electronic supplementary material.

Electronic supplementary material 1 (PDF 1130 kb)—Figure S1: Tree generated with MUSCLE alignment of sequences.

Electronic supplementary material 2 (PDF 1478 kb)—Figure S2: Tree generated with MAFFT alignment of sequences.

Electronic supplementary material 3 (PDF 1157 kb)—Figure S3: Tree generated with PRANK alignment of sequences.

Electronic supplementary material 4 (PDF 1134 kb)—Figure S4: Tree showing presence of tryptophan tetrads.

Electronic supplementary material 5 (PDF 1464 kb)—Figure S5: Trees showing the presence of the alternative tryptophan triad.

Electronic supplementary material 6 (DOCX 83 kb)—Table S1: List of all sequences used in phylogenetic analysis

## Data Availability

Data provided as Supplementary Information.
