# Supplementary figures and images for: A Case Study of Eukaryogenesis: The Evolution of Photoreception by Photolyase/Cryptochrome Proteins
Source: J Mol Evol. 2020 Sep 26;88(8):662–73. doi: 10.1007/s00239-020-09965-x (PMC7560933; doi:10.1007/s00239-020-09965-x)

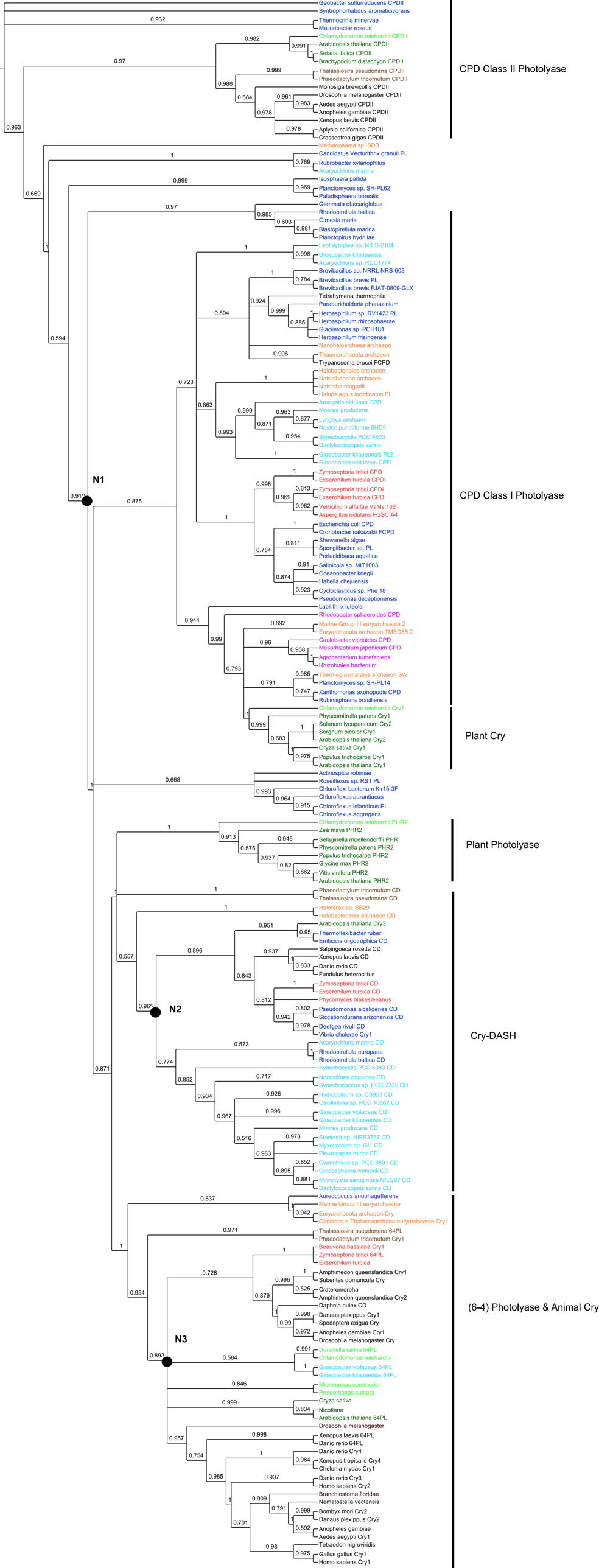

Supplement: Supplementary file 1 — Electronic supplementary material 1 (PDF 1130 kb)—Figure S1: Tree generated with MUSCLE alignment of sequences. [file 239_2020_9965_MOESM1_ESM.pdf]

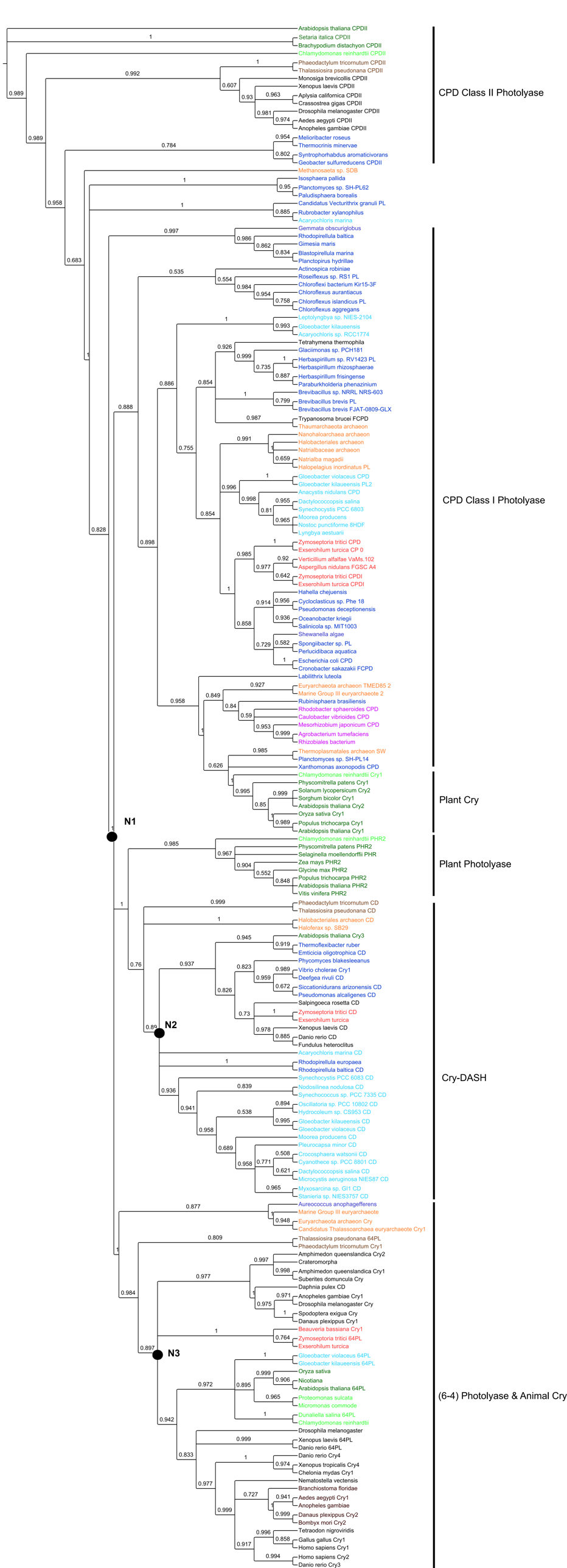

Supplement: Supplementary file 2 — Electronic supplementary material 2 (PDF 1478 kb)—Figure S2: Tree generated with MAFFT alignment of sequences. [file 239_2020_9965_MOESM2_ESM.pdf]

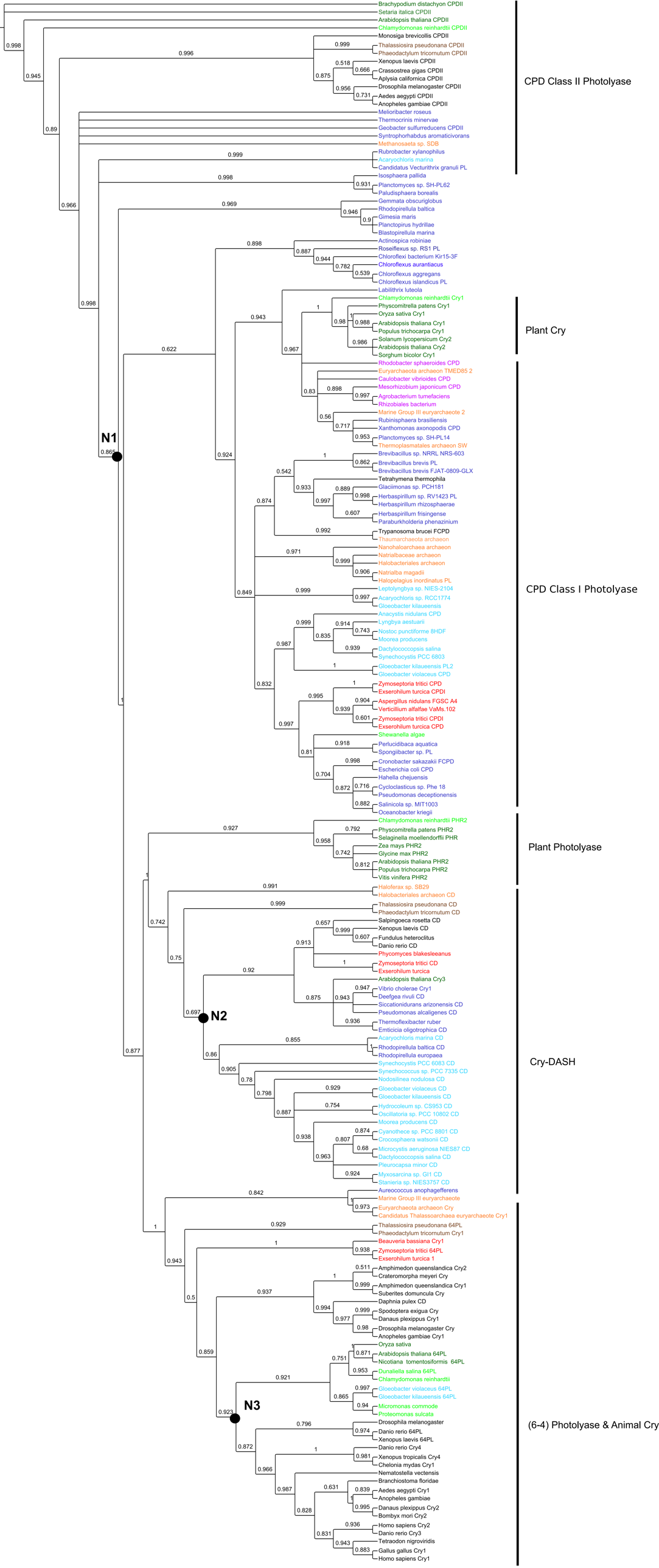

Supplement: Supplementary file 3 — Electronic supplementary material 3 (PDF 1157 kb)—Figure S3: Tree generated with PRANK alignment of sequences. [file 239_2020_9965_MOESM3_ESM.pdf]

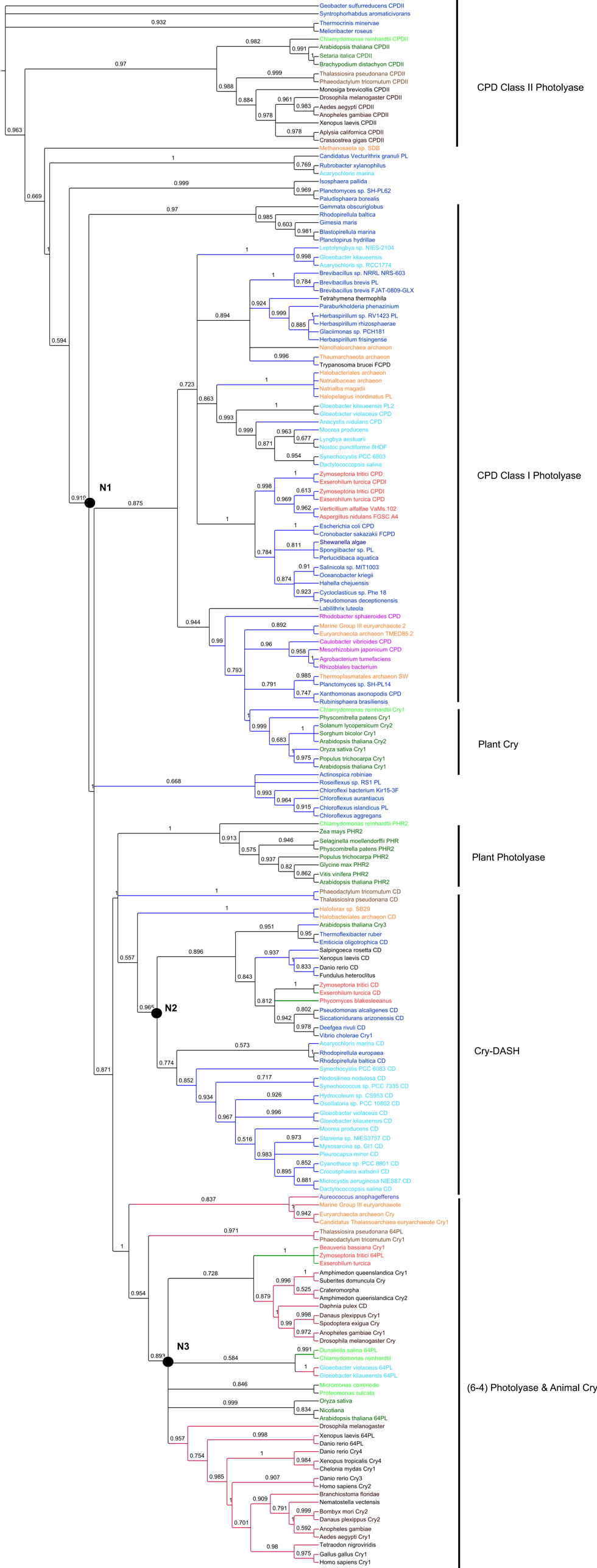

Supplement: Supplementary file 4 — Electronic supplementary material 4 (PDF 1134 kb)—Figure S4: Tree showing presence of tryptophan tetrads. [file 239_2020_9965_MOESM4_ESM.pdf]

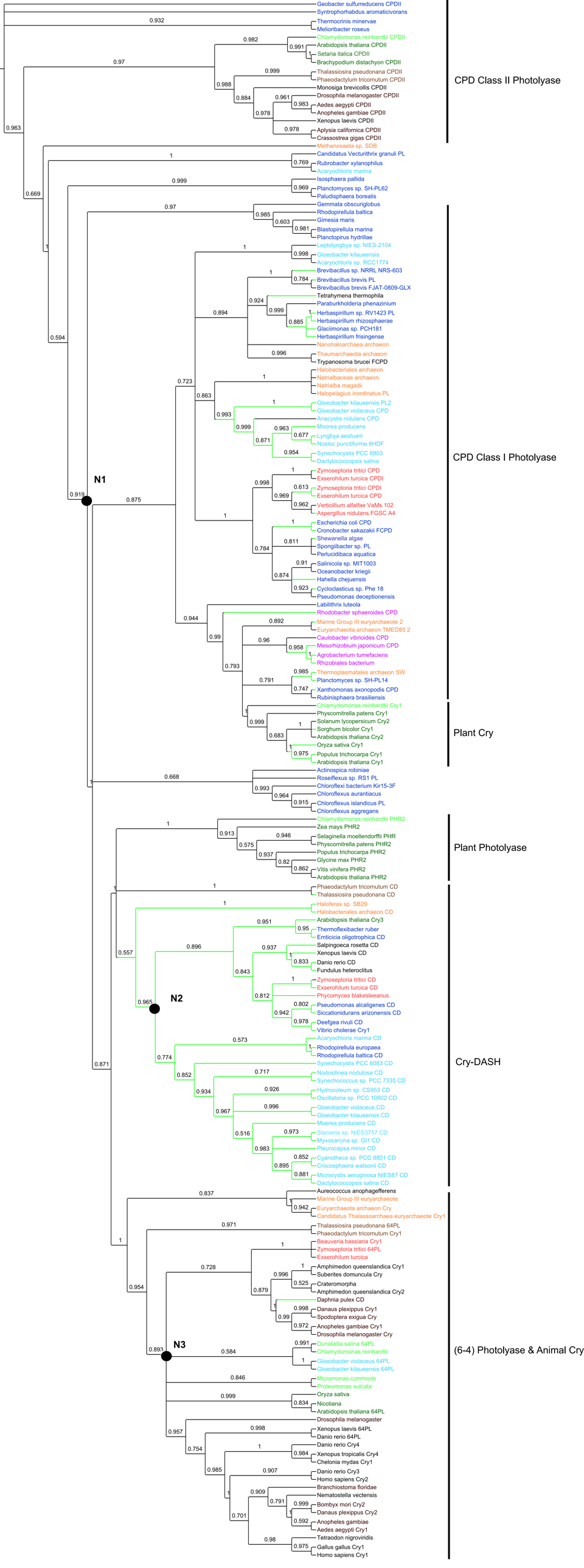

Supplement: Supplementary file 5 — Electronic supplementary material 5 (PDF 1464 kb)—Figure S5: Trees showing the presence of the alternative tryptophan triad. [file 239_2020_9965_MOESM5_ESM.pdf]
