# Supplementary material for: A Case Study of Eukaryogenesis: The Evolution of Photoreception by Photolyase/Cryptochrome Proteins
Source: J Mol Evol. 2020 Sep 26;88(8):662–73. doi: 10.1007/s00239-020-09965-x (PMC7560933; doi:10.1007/s00239-020-09965-x)
Supplement: Supplementary file 6 — Electronic supplementary material 6 (DOCX 83 kb)—Table S1: List of all sequences used in phylogenetic analysis [file 239_2020_9965_MOESM6_ESM.docx]

**Sequence database**

| **Full name** | **Species** | **Subfamily** | **Accession code** | **Annotation in tree** | **Conserved residues** | | **Tryptophans** | | | |  |
| --- | --- | --- | --- | --- | --- | --- | --- | --- | --- | --- | --- |
| [Acaryochloris marina MBIC11017](https://www.ncbi.nlm.nih.gov/Taxonomy/Browser/wwwtax.cgi?mode=Info&id=329726) | Cyanobacteria |  | WP_012161330.1 | Acaryochloris marina | M | E | - | - | - | - | 2 |
| [Acaryochloris marina MBIC11017](https://www.ncbi.nlm.nih.gov/Taxonomy/Browser/wwwtax.cgi?mode=Info&id=329726) | Cyanobacteria | CRY-DASH | WP_012162902.1, | Acaryochloris marina CD | Q | Y | G | L | N | W | 3 |
| Acaryochloris sp. RCC1774 | Cyanobacteria |  | WP_110985607.1 | Acaryochloris sp. RCC1774 | A | W | F | L | P | W |  |
| A[ctinospica robiniae DSM 44927](https://www.ncbi.nlm.nih.gov/Taxonomy/Browser/wwwtax.cgi?mode=Info&id=479430) | Actinobacteria |  | WP_034263007.1 | Actinospica robiniae | M | W | G | V | D | W |  |
| Aedes aegypti | Insect | Cry1 | XP_001655778.2 | Aedes aegypti Cry1 | H | W | W | I | S | W | 4 |
| Aedes aegypti | Insect | CPD II | XP_021698749.1 | Aedes aegypti CPDII | M | - | I | L | E | W |  |
| [Agrobacterium tumefaciens](https://www.ncbi.nlm.nih.gov/Taxonomy/Browser/wwwtax.cgi?mode=Info&id=358) | Alphaproteobacteria |  | WP_010971478.1 | Agrobacterium tumefaciens | M | W | L | I | D | W |  |
| Amphimedon queenslandica | Sponge | Cry1 | [XP_003386582.1](https://www.ncbi.nlm.nih.gov/protein/XP_003386582.1?report=genbank&log$=prottop&blast_rank=1&RID=KCGHGNVH01R) | Amphimedon queenslandica Cry1 | Q | Q | W | I | S | W | 4 |
| Amphimedon queenslandica | Sponge | Cry2 | XP_003386569.1 | Amphimedon queenslandica Cry2 | E | K | W | I | S | W | 4 |
| Synechococcus elongates PCC 7942 | Cyanobacteria | CPD -8HDF | WP_011377448.1 | Anacystis nidulans CPD | M | W | I | I | D | W | 3 |
| Anopheles gambiae str. PEST | Insect | Cry2 | XP_313179.4 | Anopheles gambiae | H | W | W | I | S | W | 4 |
| Anopheles gambiae str. PEST | Insect | Cry1 | XP_321104.4 | Anopheles gambiae Cry1 | N | W | W | L | S | W | 4 |
| Anopheles gambiae str. PEST | Insect | CPD II | XP_313925.3 | Anopheles gambiae CPD II | M | K | - | - | - | - | 2 |
| Aplysia californica | Sea Slug | CPD II | XP_012935158.1 | Aplysia californica | M | - | I | L | E | W |  |
| Arabidopsis thaliana | Plant | (6-4) PL | NP_566520.1 | Arabidopsis thaliana 64PL | H | W | F | I | H | W | 3 |
| Arabidopsis thaliana | Plant | CPD II | NP_849651.1 | Arabidopsis thaliana CPD II | M | K | - | - | - | - | 2 |
| Arabidopsis thaliana | Plant | Cry1 | NP_567341.1 | Arabidopsis thaliana Cry1 | V | Y | Q | L | P | W | 3 |
| Arabidopsis thaliana | Plant | Cry2 | NP_171935.1 | Arabidopsis thaliana Cry2 | V | Y | L | L | P | W | 3 |
| Arabidopsis thaliana | Plant | Cry3 | NP_568461.3 | Arabidopsis thaliana Cry3 | Q | Y | G | L | D | W | 3 |
| Arabidopsis thaliana | Plant | PHR2 | NP_182281.1 | Arabidopsis thaliana PHR2 | - | Y | - | - | - | - |  |
| Aspergillus nidulans FGSC A4 | Fungi |  | XP_657991.1 | Aspergillus nidulans FGSC A4 | M | F | L | I | D | W | 3 |
| Aureococcus anophagefferens | Stramenopiles |  | XP_009033826.1 | Aureococcus anophagefferens | H | G | W | L | P | W | 4 |
| bacterium HR32 | Bacteria |  | GBD29221.1 | bacterium HR32 PL | L | W | L | V | D | W | 3 |
| Beauveria bassiana | Fungi | Cry1 | PMB69295.1 | Beauveria bassiana Cry1 | H | W | Y | V | D | W | 3 |
| Blastopirellula marina | Planctomyces |  | WP_002652998.1 | Blastopirellula marina | M | T | - | - | - | - | 2 |
| Bombyx mori (domestic silkworm) | Insect | Cry 2 | NP_001182627.1 | Bombyx mori Cry2 | H | W | W | I | S | W | 4 |
| Brachypodium distachyon (stiff brome) | Viridiplantae |  | XP_003573808.1 | Brachypodium distachyon CPDII | M | S | - | - | - | - | 2 |
| Branchiostoma floridae (Florida lancelet) | Chordata |  | XP_002609501.1 | Branchiostoma floridae | H | W | W | I | S | W | 4 |
| Brevibacillus brevis | Firmicutes |  | WP_015893650.1 | Brevibacillus brevis PL | M | W | L | V | D | W | 3 |
| Brevibacillus brevis FJAT-0809-GLX | Firmicutes |  | WP_016740168.1 | Brevibacillus brevis FJAT-0809-GLX | M | W | L | V | D | W |  |
| B[revibacillus sp. NRRL NRS-603](https://www.ncbi.nlm.nih.gov/Taxonomy/Browser/wwwtax.cgi?mode=Info&id=2126351) | Firimicutes |  | WP_106779477.1 | Brevibacillus sp. NRRL NRS-603 | M | W | L | V | D | W |  |
| Candidatus Peregrinibacteria bacterium HGW-Peregrinibacteria- (groundwater metagenome) | Unclassified bacteria |  | PKL36547.1 | Candidatus Peregrinibacteria bacterium | M | E | - | - | - | - | 2 |
| Candidatus Saccharibacteria bacterium RIFCSPHIGHO2_01_FULL_45_15 | Saccharibacteria |  | [OGL22230.1](https://www.ncbi.nlm.nih.gov/protein/OGL22230.1?report=genbank&log$=prottop&blast_rank=1&RID=KWA1A6N7014) | Candidatus Saccharibacteria | L | W | W | I | D | W | 4 |
| uncultured Candidatus Thalassoarchaea euryarchaeote | Euryarchaeota |  | [ANV79670.1](https://www.ncbi.nlm.nih.gov/protein/ANV79670.1?report=genbank&log$=protalign&blast_rank=1&RID=KWA424FA01R) | Candidatus Thalassoarchaea euryarchaeote Cry1 | H | W | W | Q | N | W | 4 |
| [Candidatus Vecturithrix granuli](http://www.uniprot.org/taxonomy/1499967) | Bacteria |  | [GAK55483.1](https://www.ncbi.nlm.nih.gov/protein/GAK55483.1?report=genbank&log$=prottop&blast_rank=1&RID=KWA66PBZ015) | Candidatus Vecturithrix granuli PL | M | E | - | - | - | - | 2 |
| Caulobacter crescentus | Alphaproteobacteria |  | [WP_010919304.1](https://www.ncbi.nlm.nih.gov/protein/WP_010919304.1?report=genbank&log$=protalign&blast_rank=1&RID=KWA83HR901R) | Caulobacter crescentus CPD | M | W | M | I | D | W | 3 |
| Chelonia mydas | Eukaryote | Cry1 | XP_007054374.1 | Chelonia mydas Cry1 | H | W | W | I | S | W | 4 |
| Chlamydomonas reinhardtii | Algae |  | [XP_001698054.1](https://www.ncbi.nlm.nih.gov/protein/XP_001698054.1?report=genbank&log$=protalign&blast_rank=1&RID=KWAUY314015) | Chlamydomonas reinhardtii | H | W | Y | V | S | W | 3 |
| Chlamydomonas reinhardtii | Algae | CPD II | AAD39433.1 | Chlamydomonas reinhardtii CPDII | M | Q | - | - | - | - | 2 |
| Chlamydomonas reinhardtii | Algae |  | [XP_001701553.1](https://www.ncbi.nlm.nih.gov/protein/XP_001701553.1?report=genbank&log$=prottop&blast_rank=1&RID=KWAMM527014) | Chlamydomonas reinhardtii Cry1 | V | Y | L | L | P | W | 3 |
| Chlamydomonas reinhardtii | Algae | PHR2 | XP_001692996.1 | Chlamydomonas reinhardtii PHR2 | - | H | - | - | - | - |  |
| [Chloroflexus aggregans DSM 9485](https://www.ncbi.nlm.nih.gov/Taxonomy/Browser/wwwtax.cgi?mode=Info&id=326427) | Chlorflexi bacteria |  | WP_012616155.1 | Chloroflexus aggregans | M | W | L | I | D | W |  |
| C[hloroflexus aurantiacus J-10-fl](https://www.ncbi.nlm.nih.gov/Taxonomy/Browser/wwwtax.cgi?mode=Info&id=324602) | Chlorflexi bacteria |  | WP_012259342.1 | Chloroflexus aurantiacus | M | W | L | I | D | W |  |
| Chloroflexi bacterium Kir15-3F | Chlorflexi bacteria |  | WP_097643853.1 | Chloroflexi bacterium Kir15-3F | M | W | L | L | D | W |  |
| Chloroflexus islandicus | Chlorflexi bacteria | PL | [WP_066782878.1](https://www.ncbi.nlm.nih.gov/protein/WP_066782878.1?report=genbank&log$=prottop&blast_rank=1&RID=PT385KTG015) | Chloroflexus islandicus PL | M | W | L | I | D | W | 3 |
| Crassostrea gigas | Oyster | CPD II | XP_011422754.2 | Crassostrea gigas CPDII | M | - | I | L | E | W |  |
| Crateromorpha meyeri | Sponge | Cry | [CAZ66368.1](https://www.ncbi.nlm.nih.gov/protein/CAZ66368.1?report=genbank&log$=prottop&blast_rank=1&RID=PT3ARC2F015) | Crateromorpha meyeri Cry | N | H | W | I | G | W | 4 |
| Crocosphaera watsonii | Cyanobacteria | CRY-DASH | [WP_007308301.1](https://www.ncbi.nlm.nih.gov/protein/WP_007308301.1?report=genbank&log$=prottop&blast_rank=1&RID=PT3DTX8M014) | Crocosphaera watsonii CD | Q | Y | G | I | N | W | 3 |
| Cronobacter sakazakii | Gammaproteobacteria | PL | [WP_012125345.1](https://www.ncbi.nlm.nih.gov/protein/WP_012125345.1?report=genbank&log$=prottop&blast_rank=1&RID=PT3FHPT5014) | Cronobacter sakazakii FCPD | M | W | L | V | D | W | 3 |
| cyanobacterium TDX16 | Cyanobacteria |  | [OWY73171.1](https://www.ncbi.nlm.nih.gov/protein/OWY73171.1?report=genbank&log$=protalign&blast_rank=1&RID=KWAMM527014) | cyanobacterium TDX16 | M | W | L | I | D | W | 2 |
| Cyanothece sp. PCC 8801 | Cyanobacteria | CRY-DASH | [WP_012594061.1](https://www.ncbi.nlm.nih.gov/protein/WP_012594061.1?report=genbank&log$=prottop&blast_rank=1&RID=PT3J3DWW014) | Cyanothece sp. PCC 8801 CD | Q | Y | G | I | N | W | 3 |
| Cycloclasticus sp. Phe 18 | Gammaproteobacteria | PL | [KXJ43476.1](https://www.ncbi.nlm.nih.gov/protein/KXJ43476.1?report=genbank&log$=prottop&blast_rank=1&RID=PT3M3263014) | Cycloclasticus sp. Phe 18 | M | W | L | I | D | W | 3 |
| Dactylococcopsis salina PCC 8305 | Cyanobacteria | CRY-DASH | [WP_015229255.1](https://www.ncbi.nlm.nih.gov/protein/WP_015229255.1?report=genbank&log$=prottop&blast_rank=1&RID=PT3NMC9E015) | Dactylococcopsis salina CD | Q | Y | G | I | D | W | 3 |
| [Dactylococcopsis salina PCC 8305](https://www.ncbi.nlm.nih.gov/Taxonomy/Browser/wwwtax.cgi?mode=Info&id=13035) | Cyanobacteria |  | WP_015229213.1 | Dactylococcopsis salina | M | W | I | I | N | W |  |
| Danaus plexippus | Insect | Cry-1 | [AAX58599.1](https://www.ncbi.nlm.nih.gov/protein/AAX58599.1?report=genbank&log$=prottop&blast_rank=1&RID=PT345KWN015) | Danaus plexippus Cry1 | N | W | W | L | S | W | 4 |
| Danaus plexippus | Insect | Cry-2 | A[BA62409.1](https://www.ncbi.nlm.nih.gov/protein/ABA62409.1?report=genbank&log$=prottop&blast_rank=1&RID=PT35UY1K014) | Danaus plexippus Cry2 | H | W | W | I | S | W | 4 |
| Danio rerio | Fish | Cry-5 | [NP_571863.1](https://www.ncbi.nlm.nih.gov/protein/NP_571863.1?report=genbank&log$=prottop&blast_rank=1&RID=PT31JWN1014) | Danio rerio 64PL | H | W | W | I | S | W | 4 |
| Danio rerio | Fish | Cry-3 | BAA96850.1 | Danio rerio Cry3 | H | W | W | I | S | W | 4 |
| Danio rerio | Fish | Cry-4 | [NP_571862.1](https://www.ncbi.nlm.nih.gov/protein/NP_571862.1?report=genbank&log$=prottop&blast_rank=1&RID=PT307XAB014) | Danio rerio CRY4 | H | W | W | I | S | W | 4 |
| Danio rerio | Fish | CRY-DASH | [NP_991249.1](https://www.ncbi.nlm.nih.gov/protein/NP_991249.1?report=genbank&log$=prottop&blast_rank=1&RID=PT32V151015) | Danio rerio CD | Q | Y | G | L | D | W | 3 |
| Daphnia pulex | Flea | (6-4) PL | [EFX77441.1](https://www.ncbi.nlm.nih.gov/protein/EFX77441.1?report=genbank&log$=prottop&blast_rank=1&RID=PT3RAM9M015) | Daphnia pulex | N | W | W | L | N | W | 4 |
| Deefgea rivuli | Betaproteobacteria | CRY-DASH | [WP_027467886.1](https://www.ncbi.nlm.nih.gov/protein/WP_027467886.1?report=genbank&log$=prottop&blast_rank=1&RID=PT3TRFTA015) | Deefgea rivuli CD | Q | Y | Q | C | D | W | 2 |
| delta proteobacterium ML8_D | Deltaproteobacteria |  | [OPL10888.1](https://www.ncbi.nlm.nih.gov/protein/OPL10888.1?report=genbank&log$=protalign&blast_rank=1&RID=KWAMM527014) | delta proteobacterium ML8 D | M | E | - | - | - | - | 2 |
| Deltaproteobacteria bacterium CG07_land_8_20_14_0_80_60_11 | Deltaproteobacteria | PL | [PIU54519.1](https://www.ncbi.nlm.nih.gov/protein/PIU54519.1?report=genbank&log$=prottop&blast_rank=1&RID=PT3VU1FZ014) | Deltaproteobacteria bacterium | M | A | - | - | - | - | 2 |
| Drosophila melanogaster | Insect | (6-4) PL | [BAA12067.1](https://www.ncbi.nlm.nih.gov/protein/BAA12067.1?report=genbank&log$=prottop&blast_rank=1&RID=PT2M1E9E014) | Drosophila melanogaster | H | W | W | I | S | W | 4 |
| Drosophila melanogaster | Insect | CPD like | [BAA05042.1](https://www.ncbi.nlm.nih.gov/protein/BAA05042.1?report=genbank&log$=prottop&blast_rank=1&RID=PT2PZ163015) | Drosophila melanogaster CPDII | M | A | - | - | - | - | 2 |
| Drosophila melanogaster | Insect | Cry | NP_732407.1 | Drosophila melanogaster Cry | N | W | W | Q | S | W | 4 |
| Dunaliella salina | Algae | (6-4) PL | AAX56342.1 | Dunaliella salina 64PL | H | W | Y | L | S | W | 3 |
| Emticicia oligotrophica | Flavobacteria | CRY-DASH | [WP_015028983.1](https://www.ncbi.nlm.nih.gov/protein/WP_015028983.1?report=genbank&log$=prottop&blast_rank=1&RID=PT3XS20W014) | Emticicia oligotrophica CD | Q | Y | G | I | D | W | 3 |
| Escherichia coli | Gammaproteobacteria | PL | [WP_032176081.1](https://www.ncbi.nlm.nih.gov/protein/WP_032176081.1?report=genbank&log$=prottop&blast_rank=1&RID=PT3ZJFJ7015) | Escherichia coli CPD | M | W | L | I | D | W | 3 |
| Euryarchaeota archaeon TMED85 | Archaea | (6-4) PL | [OUU99058.1](https://www.ncbi.nlm.nih.gov/protein/OUU99058.1?report=genbank&log$=prottop&blast_rank=1&RID=PT41F9XG014) | Euryarchaeota archaeon Cry | H | W | W | Q | N | W | 4 |
| Euryarchaeota archaeon TMED85 | Archaea | PL | [OUU99940.1](https://www.ncbi.nlm.nih.gov/protein/OUU99940.1?report=genbank&log$=prottop&blast_rank=1&RID=PT43NJMB015) | Euryarchaeota archaeon TMED85 2 | M | W | L | I | D | W | 3 |
| Exserohilum turcica Et28A | Fungi | CRY-DASH | [XP_008021585.1](https://www.ncbi.nlm.nih.gov/protein/XP_008021585.1?report=genbank&log$=protalign&blast_rank=1&RID=RBHNNE33014) | Exserohilum turcica CD | Q | Y | K | I | D | W | 3 |
| [Exserohilum turcica Et28A](https://www.ncbi.nlm.nih.gov/Taxonomy/Browser/wwwtax.cgi?mode=Info&id=671987) | Fungi |  | [XP_008026696.1](https://www.ncbi.nlm.nih.gov/protein/XP_008026696.1?report=genbank&log$=protalign&blast_rank=1&RID=RBHK6A6P014) | Exserohilum turcica CPDI | M | - | L | L | D | Y | 2 |
| Exserohilum turcica Et28A | Fungi | Cry-2 | [XP_008021998.1](https://www.ncbi.nlm.nih.gov/protein/XP_008021998.1?report=genbank&log$=prottop&blast_rank=1&RID=PT2T22CH014) | Exserohilum turcica | H | W | Y | I | D | W | 3 |
| Exserohilum turcica Et28A | Fungi | CPD I | [XP_008025248.1](https://www.ncbi.nlm.nih.gov/protein/XP_008025248.1?report=genbank&log$=prottop&blast_rank=1&RID=PT2VM6W3015) | Exserohilum turcica CPDI | M | F | L | L | D | W | 3 |
| Fundulus heteroclitus | Fish | CRY-DASH | [XP_012735645.1](https://www.ncbi.nlm.nih.gov/protein/XP_012735645.1?report=genbank&log$=prottop&blast_rank=1&RID=PT45SJVN015) | Fundulus heteroclitus | Q | Y | G | L | D | W | 3 |
| Gallus gallus | Bird | Cry-1 | [NP_989576.1](https://www.ncbi.nlm.nih.gov/protein/NP_989576.1?report=genbank&log$=prottop&blast_rank=1&RID=PT47N061014) | Gallus gallus Cry1 | H | W | W | I | S | W | 4 |
| Gammaproteobacteria bacterium TMED243 | Gammaproteobacteria | PL | [OUX13911.1](https://www.ncbi.nlm.nih.gov/protein/OUX13911.1?report=genbank&log$=prottop&blast_rank=1&RID=PT4BMB7W015) | Gammaproteobacteria TMED243 | H | W | W | Q | N | W | 4 |
| Gammaproteobacteria bacterium SG8 11 | Gammaproteobacteria | PL | [KPJ96232.1](https://www.ncbi.nlm.nih.gov/protein/KPJ96232.1?report=genbank&log$=prottop&blast_rank=1&RID=PT492XHR015) | Gammaproteobacteria bacterium SG8 11 | M | K | - | - | - | - | 2 |
| Gemmata obscuriglobus | Planctomyces | PL | [WP_010044050.1](https://www.ncbi.nlm.nih.gov/protein/WP_010044050.1?report=genbank&log$=prottop&blast_rank=1&RID=PT4DACTR015) | Gemmata obscuriglobus | M | A | - | - | - | - | 2 |
| Geobacter sulfurreducens | Deltaproteobacteria | PL | [WP_010943456.1](https://www.ncbi.nlm.nih.gov/protein/WP_010943456.1?report=genbank&log$=prottop&blast_rank=1&RID=PT4FN5YZ014) | Geobacter sulfurreducens CPDII | M | S | - | - | - | - | 2 |
| [Gimesia maris DSM 8797](https://www.ncbi.nlm.nih.gov/Taxonomy/Browser/wwwtax.cgi?mode=Info&id=344747) | Planctomyces |  | WP_002645708.1 | Gimesia maris | M | - | I | L | H | W |  |
| G[laciimonas sp. PCH181](https://www.ncbi.nlm.nih.gov/Taxonomy/Browser/wwwtax.cgi?mode=Info&id=2133943) | Betaproteobacteria |  | WP_108440310.1 | Glaciimonas sp. PCH181 | M | W | G | I | D | W |  |
| [Gloeobacter kilaueensis JS1](https://www.ncbi.nlm.nih.gov/Taxonomy/Browser/wwwtax.cgi?mode=Info&id=1183438) | Cyanobacteria |  | [WP_023175030.1](https://www.ncbi.nlm.nih.gov/protein/WP_023175030.1?report=genbank&log$=prottop&blast_rank=1&RID=P5XW4DWA01R) | Gloeobacter kilaueensis | A | S | L | L | P | W | 3 |
| [Gloeobacter kilaueensis JS1](https://www.ncbi.nlm.nih.gov/Taxonomy/Browser/wwwtax.cgi?mode=Info&id=1183438) | Cyanobacteria | CRY-DASH | [WP_023173156.1](https://www.ncbi.nlm.nih.gov/protein/WP_023173156.1?report=genbank&log$=prottop&blast_rank=1&RID=P5XS9SHP014) | Gloeobacter kilaueensis CD | Q | Y | G | I | D | W | 3 |
| [Gloeobacter kilaueensis JS1](https://www.ncbi.nlm.nih.gov/Taxonomy/Browser/wwwtax.cgi?mode=Info&id=1183438) | Cyanobacteria |  | [WP_023172734.1](https://www.ncbi.nlm.nih.gov/protein/WP_023172734.1?report=genbank&log$=prottop&blast_rank=1&RID=P5Y2G8DA01R) | Gloeobacter kilaueensis 64PL | H | W | W | L | S | W | 4 |
| [Gloeobacter kilaueensis JS1](https://www.ncbi.nlm.nih.gov/Taxonomy/Browser/wwwtax.cgi?mode=Info&id=1183438) | Cyanobacteria |  | [WP_023172026.1](https://www.ncbi.nlm.nih.gov/protein/WP_023172026.1?report=genbank&log$=prottop&blast_rank=1&RID=P5XU2KRU01R) | Gloeobacter kilaueensis PL2 | M | W | L | L | P | Y | 1 |
| Gloeobacter violaceus PCC 7421 | Cyanobacteria |  | [WP_011141747.1](https://www.ncbi.nlm.nih.gov/protein/WP_011141747.1?report=genbank&log$=prottop&blast_rank=1&RID=P5XZH97U01R) | Gloeobacter violaceus 64PL | H | W | W | V | S | W | 4 |
| Gloeobacter violaceus PCC 7421 | Cyanobacteria |  | WP_011140786.1 | Gloeobacter violaceus CPD | M | W | L | L | P | Y | 2 |
| Gloeobacter violaceus PCC 7421 | Cyanobacteria | CRY-DASH | WP_011140837.1 | Gloeobacter violaceus CD | Q | Y | G | I | H | W | 3 |
| Glycine max | Plant | PHR2 | [XP_003536113.1](https://www.ncbi.nlm.nih.gov/protein/XP_003536113.1?report=genbank&log$=prottop&blast_rank=1&RID=PT4HHB2C015) | Glycine max PHR2 | - | Y | - | - | - | - |  |
| Hahella chejuensis | Marine bacteria | PL | [WP_011395654.1](https://www.ncbi.nlm.nih.gov/protein/WP_011395654.1?report=genbank&log$=prottop&blast_rank=1&RID=PT4KJPMM014) | Hahella chejuensis | M | W | L | L | D | W | 3 |
| Halobacteriales archaeon QH_7_68_42 | Archaea | PL | [PSP54631.1](https://www.ncbi.nlm.nih.gov/protein/PSP54631.1?report=genbank&log$=prottop&blast_rank=1&RID=PT4N34VJ015) | Halobacteriales archaeon | M | W | M | I | D | W | 3 |
| Halobacteriales archaeon QS_5_70_15 | Archaea | CRY-DASH | [PSQ10710.1](https://www.ncbi.nlm.nih.gov/protein/PSQ10710.1?report=genbank&log$=prottop&blast_rank=1&RID=PT4S06G7015) | Halobacteriales archaeon CD | Q | Y | E | I | D | W | 3 |
| Haloferax sp. SB29 | Archaea | CRY-DASH | WP_058572176.1 | Haloferax sp. SB29 | Q | Y | R | L | D | W | 3 |
| Halopelagius inordinatus | Archaea | PL | [WP_092891184.1](https://www.ncbi.nlm.nih.gov/protein/WP_092891184.1?report=genbank&log$=prottop&blast_rank=1&RID=PT4Y6EZV015) | Halopelagius inordinatus PL | M | W | L | L | D | W | 3 |
| [Herbaspirillum frisingense GSF30](https://www.ncbi.nlm.nih.gov/Taxonomy/Browser/wwwtax.cgi?mode=Info&id=864073) | Betaproteobacteria |  | WP_006463571.1 | Herbaspirillum frisingense | M | W | G | I | D | W |  |
| [Herbaspirillum rhizosphaerae](https://www.ncbi.nlm.nih.gov/Taxonomy/Browser/wwwtax.cgi?mode=Info&id=346179) | Betaproteobacteria |  | WP_050476644.1 | Herbaspirillum rhizosphaerae | M | W | G | I | D | W |  |
| Herbaspirillum sp. RV1423 | Betaproteobacteria |  | [WP_034292518.1](https://www.ncbi.nlm.nih.gov/protein/WP_034292518.1?report=genbank&log$=prottop&blast_rank=1&RID=PT5063AR015) | Herbaspirillum sp. RV1423 PL | M | W | G | I | D | W | 3 |
| Homo sapiens | Mammal | Cry | NP_004066.1 | Homo sapiens Cry1 | H | W | W | I | S | W | 4 |
| Homo sapiens | Mammal | Cry | BAG64048.1 | Homo sapiens Cry2 | H | W | W | V | S | W | 4 |
| Hydrocoleum sp. CS-953 | Cyanobacteria | CRY-DASH | WP_094671595.1 | Hydrocoleum sp. CS953 CD | Q | Y | G | I | N | W | 3 |
| Ignavibacteria bacterium CG_4_8_14_3_um_filter_37_9 | FCB group bacteria | PL | [PIW99777.1](https://www.ncbi.nlm.nih.gov/protein/PIW99777.1?report=genbank&log$=prottop&blast_rank=1&RID=PT52UXGU015) | Ignavibacteria bacterium PL2 | M | E | - | - | - | - | 2 |
| [Isosphaera pallida ATCC 43644](https://www.ncbi.nlm.nih.gov/Taxonomy/Browser/wwwtax.cgi?mode=Info&id=575540) | Bacteria |  | WP_013563604.1 | Isosphaera pallida | M | - | I | L | E | W |  |
| [Labilithrix luteola](https://www.ncbi.nlm.nih.gov/Taxonomy/Browser/wwwtax.cgi?mode=Info&id=1391654) | Deltaproteobacteria |  | AKU98937.1 | Labilithrix luteola | M | W | L | V | D | F |  |
| Leptolyngbya sp. NIES-2104 | Cyanobacteria |  | WP_058994625.1 | Leptolyngbya sp. NIES-2104 | A | M | G | M | D | W |  |
| Lyngbya aestuarii | Cyanobacteria | PL | [WP_023067321.1](https://www.ncbi.nlm.nih.gov/protein/WP_023067321.1?report=genbank&log$=prottop&blast_rank=1&RID=PT54FBSW015) | Lyngbya aestuarii | M | W | M | I | N | W | 3 |
| Marine Group III euryarchaeote CG-Epi1 | Archaea | (6-4)PL | [OIR17544.1](https://www.ncbi.nlm.nih.gov/protein/OIR17544.1?report=genbank&log$=prottop&blast_rank=1&RID=PT563GE3015) | Marine Group III euryarchaeote | H | W | W | Q | H | W | 4 |
| Marine Group III euryarchaeote CG-Epi2 | Archaea | PL | OIR22355.1 | Marine Group III euryarchaeote 2 | M | W | L | L | D | W | 3 |
| Mariniblastus fucicola | Planctomyces |  | WP_075084086.1 | Mariniblastus fucicola | M | W | L | I | D | W |  |
| Melioribacter roseus | FCB group bacteria | PL | [WP_014856093.1](https://www.ncbi.nlm.nih.gov/protein/WP_014856093.1?report=genbank&log$=prottop&blast_rank=1&RID=PT5A7CGK015) | Melioribacter roseus | M | N | - | - | - | - | 2 |
| Mesorhizobium japonicum | Alphaproteobacteria | PL | [WP_010915359.1](https://www.ncbi.nlm.nih.gov/protein/WP_010915359.1?report=genbank&log$=prottop&blast_rank=1&RID=PT5BEHJ2014) | Mesorhizobium japonicum CPD | M | W | M | I | D | W | 3 |
| Methanosaeta | Archaea | PL | [KQC15385.1](https://www.ncbi.nlm.nih.gov/protein/KQC15385.1?report=genbank&log$=prottop&blast_rank=1&RID=PT5DYAXP015) | Methanosaeta sp. SDB | M | E | - | - | - | - | 2 |
| Microcystis aeruginosa NIES87 | Cyanobacteria | CRY-DASH | [WP_104396464.1](https://www.ncbi.nlm.nih.gov/protein/WP_104396464.1?report=genbank&log$=prottop&blast_rank=1&RID=PT5FB47M015) | Microcystis aeruginosa NIES87 PL | Q | Y | G | I | D | W | 3 |
| Micromonas commode | Algae | (6-4) PL | [XP_002508785.1](https://www.ncbi.nlm.nih.gov/protein/XP_002508785.1?report=genbank&log$=prottop&blast_rank=1&RID=PT5JC5WH015) | Micromonas commode | H | W | F | V | H | W | 3 |
| Monosiga brevicollis MX1 | Choanoflagellate | CPD II | [XP_001746666.1](https://www.ncbi.nlm.nih.gov/protein/XP_001746666.1?report=genbank&log$=prottop&blast_rank=1&RID=PT5M91TT015) | Monosiga Brevicollis CPDII | M | - | I | L | E | W | 3 |
| Moorea producens | Cyanobacteria | PL | WP_070391697.1 | Moorea producens | M | W | I | I | N | W | 3 |
| Moorea producens | Cyanobacteria | CRY-DASH | WP_083305032.1 | Moorea producens CD | Q | Y | G | I | N | W | 3 |
| Myxosarcina sp. GI1 | Cyanobacteria | CRY-DASH | [WP_036482731.1](https://www.ncbi.nlm.nih.gov/protein/WP_036482731.1?report=genbank&log$=prottop&blast_rank=1&RID=PT5SVU9S015) | Myxosarcina sp. GI1 CD | Q | Y | G | I | N | W | 3 |
| Nanohaloarchaea archaeon SW_7_43_1 | Archaea | PL | PSG99464.1 | Nanohaloarchaea archaeon | M | W | W | L | D | W | 3 |
| Natrialba magadii | Halobacteria | PL | [WP_004214180.1](https://www.ncbi.nlm.nih.gov/protein/WP_004214180.1?report=genbank&log$=prottop&blast_rank=1&RID=PT5XTNJH014) | Natrialba magadii | M | W | M | L | D | W | 3 |
| Natrialbaceae archaeon JW/NM-HA 15 | Archaea | PL | [WP_086886690.1](https://www.ncbi.nlm.nih.gov/protein/WP_086886690.1?report=genbank&log$=prottop&blast_rank=1&RID=PT5ZKWNR015) | Natrialbaceae archaeon | M | W | L | L | D | W | 3 |
| Nematostella vectensis | Anemone | Cry-1 | [XP_001623146.1](https://www.ncbi.nlm.nih.gov/protein/XP_001623146.1?report=genbank&log$=prottop&blast_rank=1&RID=PT61JFBG015) | Nematostella vectensis | V | W | W | I | S | W | 4 |
| Nicotiana tomentosiformis | Plant | (6-4) PL | [XP_018625566.1](https://www.ncbi.nlm.nih.gov/protein/XP_018625566.1?report=genbank&log$=prottop&blast_rank=1&RID=PT63YNHW014) | Nicotiana tomentosiformis 64PL | H | W | F | V | H | W | 3 |
| Nodosilinea nodulosa | Cyanobacteria | CRY-DASH | [WP_017299930.1](https://www.ncbi.nlm.nih.gov/protein/WP_017299930.1?report=genbank&log$=prottop&blast_rank=1&RID=PT65YBF0015) | Nodosilinea nodulosa CD | Q | Y | G | I | D | W | 3 |
| Nostoc punctiforme | Cyanobacteria | 8-HDF | [WP_012408666.1](https://www.ncbi.nlm.nih.gov/protein/WP_012408666.1?report=genbank&log$=prottop&blast_rank=1&RID=PT67BJW3015) | Nostoc punctiforme 8HDF | M | W | L | I | N | P | 2 |
| Nostoc sp. 3335mG | Cyanobacteria |  | WP_110157002.1 | Nostoc sp. 3335mG | M | W | L | I | D | W |  |
| Oceanobacter kriegii | Gammaproteobacteria | PL | [WP_028294900.1](https://www.ncbi.nlm.nih.gov/protein/WP_028294900.1?report=genbank&log$=prottop&blast_rank=1&RID=PT693G5N015) | Oceanobacter kriegii | M | W | L | I | D | W | 3 |
| Opitutae bacterium TousC10FEB | Verrucomicrobia | PL | [PAW87714.1](https://www.ncbi.nlm.nih.gov/protein/PAW87714.1?report=genbank&log$=prottop&blast_rank=1&RID=PT6ASAB3015) | Opitutae bacterium TousC10FEB | M | W | R | I | S | W | 3 |
| Oryza sativa Japonica Group | Plant | (6-4) PL | [EEE56527.1](https://www.ncbi.nlm.nih.gov/protein/EEE56527.1?report=genbank&log$=prottop&blast_rank=1&RID=PT6C46TS015) | Oryza sativa | H | W | F | I | H | W | 3 |
| Oryza sativa Japonica Group | Plant | Cry1 | [XP_015625401.1](https://www.ncbi.nlm.nih.gov/protein/XP_015625401.1?report=genbank&log$=prottop&blast_rank=1&RID=PT6DTZE5015) | Oryza sativa Cry1 | V | Y | Q | L | P | W | 3 |
| Oscillatoria sp. PCC 10802 | Cyanobacteria | CRY-DASH | [WP_017720452.1](https://www.ncbi.nlm.nih.gov/protein/WP_017720452.1?report=genbank&log$=prottop&blast_rank=1&RID=PT6FJDXN015) | Oscillatoria sp. PCC 10802 CD | Q | Y | G | I | N | W | 3 |
| Paludisphaera borealis | Planctomyces |  | WP_076350111.1 | Paludisphaera borealis | M | - | I | L | E | W |  |
| P[araburkholderia phenazinium](https://www.ncbi.nlm.nih.gov/Taxonomy/Browser/wwwtax.cgi?mode=Info&id=60549) | Betaproteobacteria |  | WP_074297006.1 | Paraburkholderia phenazinium | M | W | G | I | D | W |  |
| Perlucidibaca | Gammaproteobacteria |  | [WP_094078696.1](https://www.ncbi.nlm.nih.gov/protein/WP_094078696.1?report=genbank&log$=protalign&blast_rank=1&RID=KWAUY314015) | Perlucidibaca aquatica | M | W | F | I | D | W | 3 |
| Phaeodactylum tricornutum CCAP 1055/1 | Marine plankton diatom | CD | [XP_002178889.1](https://www.ncbi.nlm.nih.gov/protein/XP_002178889.1?report=genbank&log$=protalign&blast_rank=1&RID=RB4DZPK1014) | Phaeodactylum tricornutum CD | Q | A | N | H | D | W | 3 |
| Phaeodactylum tricornutum CCAP 1055/1 | Marine plankton diatom |  | [XP_002180071.1](https://www.ncbi.nlm.nih.gov/protein/XP_002180071.1?report=genbank&log$=protalign&blast_rank=1&RID=RB4CPWBZ015) | Phaeodactylum tricornutum CPDII | M | E | - | - | - | - | 2 |
| Phaeodactylum tricornutum CCAP 1055/1 | Marine plankton diatom | Cry1 | [XP_002180095.1](https://www.ncbi.nlm.nih.gov/protein/XP_002180095.1?report=genbank&log$=protalign&blast_rank=1&RID=RB4FRGM6014) | Phaeodactylum tricornutum Cry1 | H | W | W | Q | S | W | 4 |
| Phycomyces blakesleeanus NRRL 1555(-) | Fungi |  | [XP_018298182.1](https://www.ncbi.nlm.nih.gov/protein/XP_018298182.1?report=genbank&log$=protalign&blast_rank=1&RID=RB4N03NN015) | Phycomyces blakesleeanus | Q | Y | Y | I | D | W | 3 |
| Physcomitrella patens | Moss |  | [XP_024380321.1](https://www.ncbi.nlm.nih.gov/protein/XP_024380321.1?report=genbank&log$=protalign&blast_rank=1&RID=RB4SNXEK015) | Physcomitrella patens Cry1 | V | Y | Q | L | P | W | 3 |
| Physcomitrella patens | Moss | PHR2 | [XP_024385627.1](https://www.ncbi.nlm.nih.gov/protein/XP_024385627.1?report=genbank&log$=protalign&blast_rank=2&RID=KWAGRD6501R) | Physcomitrella patens PHR2 | - | Y | - | - | - | - |  |
| Planctomyces sp. SCGC AG-212-M04 | Planctomyces |  | [OAI56716.1](https://www.ncbi.nlm.nih.gov/protein/OAI56716.1?report=genbank&log$=prottop&blast_rank=1&RID=KWAMM527014) | Planctomyces sp. SCGC AG212M04 | M | W | L | I | P | W | 3 |
| Planctomyces sp. SH-PL14 | Planctomyces |  | WP_075091512.1 | Planctomyces sp. SH-PL14 | M | W | L | Q | S | W |  |
| Planctomyces sp. SH-PL62 | Planctomyces |  | WP_082858287.1 | Planctomyces sp. SH-PL62 | M | - | I | L | E | W |  |
| P[lanctomycetaceae bacterium](https://www.ncbi.nlm.nih.gov/Taxonomy/Browser/wwwtax.cgi?mode=Info&id=2026779) | Planctomyces |  | PHY02506.1 | Planctomycetaceae bacterium | M | W | R | V | S | W |  |
| P[lanctopirus hydrillae](https://www.ncbi.nlm.nih.gov/Taxonomy/Browser/wwwtax.cgi?mode=Info&id=1841610) | Planctomyces |  | WP_068850576.1 | Planctopirus hydrillae | M | - | V | L | E | W |  |
| Pleurocapsa minor | Cyanobacteria | CRY-DASH | [WP_015144761.1](https://www.ncbi.nlm.nih.gov/protein/WP_015144761.1?report=genbank&log$=protalign&blast_rank=1&RID=RB4TYG39015) | Pleurocapsa minor CD | Q | Y | G | I | N | W | 3 |
| Populus trichocarpa | Plant | Cry1 | [XP_002307379.1](https://www.ncbi.nlm.nih.gov/protein/XP_002307379.1?report=genbank&log$=protalign&blast_rank=1&RID=RB4KEJR7014) | Populus trichocarpa Cry1 | V | Y | Q | L | P | W | 3 |
| Populus trichocarpa | Plant | PHR2 | [XP_006375454.2](https://www.ncbi.nlm.nih.gov/protein/XP_006375454.2?report=genbank&log$=protalign&blast_rank=1&RID=RB4HWC88014) | Populus trichocarpa PHR2 | - | Y | - | - | - | - |  |
| Proteomonas_sulcata | Algae | Cry1 |  | Proteomonas_sulcata | H | W | W | V | S | W | 3 |
| [Pseudanabaena sp. 'Roaring Creek'](https://www.ncbi.nlm.nih.gov/Taxonomy/Browser/wwwtax.cgi?mode=Info&id=1681830) | Cyanobacteria |  | WP_055075976.1 | Pseudanabaena sp. 'Roaring Creek' | M | W | I | I | N | W |  |
| Pseudomonas alcaligenes | Gammaproteobacteria | CD | [WP_043243840.1](https://www.ncbi.nlm.nih.gov/protein/WP_043243840.1?report=genbank&log$=protalign&blast_rank=1&RID=RB4ZM0AF015) | Pseudomonas alcaligenes CD | Q | Y | E | Q | D | W | 2 |
| Pseudomonas deceptionensis | Gammaproteobacteria |  | [WP_048360113.1](https://www.ncbi.nlm.nih.gov/protein/WP_048360113.1?report=genbank&log$=protalign&blast_rank=1&RID=RB519GYC014) | Pseudomonas deceptionensis | M | W | L | I | D | W | 3 |
| Rhizobiales bacterium | Alphaproteobacteria |  | WP_113092897.1 | Rhizobiales bacterium | M | W | L | I | D | W |  |
| Rhodobacter sphaeroides | Alphaproteobacteria |  | [WP_023003558.1](https://www.ncbi.nlm.nih.gov/protein/WP_023003558.1?report=genbank&log$=protalign&blast_rank=1&RID=RB53P258015) | Rhodobacter sphaeroides CPD | L | W | L | T | D | W | 3 |
| Rhodopirellula baltica | Planctomyces |  | [WP_037229716.1](https://www.ncbi.nlm.nih.gov/protein/WP_037229716.1?report=genbank&log$=protalign&blast_rank=1&RID=RB55TWTK014) | Rhodopirellula baltica | M | Q | - | - | - | - | 2 |
| Rhodopirellula baltica | Planctomyces |  | WP_007331578.1 | Rhodopirellula baltica | Q | Y | G | I | D | W |  |
| Rhodopirellula europaea | Planctomyces |  | WP_008656794.1 | Rhodopirellula europaea | Q | Y | G | I | D | W |  |
| Roseiflexus sp. RS-1 | Chloroflexi |  | [WP_011958146.1](https://www.ncbi.nlm.nih.gov/protein/WP_011958146.1?report=genbank&log$=protalign&blast_rank=1&RID=RBJ4B6AT015) | Roseiflexus sp. RS1 PL | M | W | L | I | D | W | 3 |
| Rubinisphaera brasiliensis | Planctomyces |  | WP_013629804.1 | Rubinisphaera brasiliensis | M | W | R | I | H | W |  |
| Rubrobacter xylanophilus | Actinobactera |  | [WP_011563651.1](https://www.ncbi.nlm.nih.gov/protein/WP_011563651.1?report=genbank&log$=protalign&blast_rank=1&RID=RBJ2189V015) | Rubrobacter xylanophilus | M | R | - | - | - | - | 2 |
| Saccharomyces cerevisiae YJM1304 | Yeast |  | [AJT93751.1](https://www.ncbi.nlm.nih.gov/protein/AJT93751.1?report=genbank&log$=protalign&blast_rank=1&RID=RBHZUHS2015) | Saccharomyces cerevisiae | M | F | L | I | D | W | 3 |
| Salinicola sp. MIT1003 | Gammaproteobacteria |  | [WP_071230740.1](https://www.ncbi.nlm.nih.gov/protein/WP_071230740.1?report=genbank&log$=protalign&blast_rank=1&RID=RBHY3CEV014) | Salinicola sp. MIT1003 | M | W | L | L | D | W | 3 |
| Salpingoeca rosetta | Choanoflagellates i | CRY-DASH | [XP_004989008.1](https://www.ncbi.nlm.nih.gov/protein/XP_004989008.1?report=genbank&log$=protalign&blast_rank=1&RID=RBHW8P2G014) | Salpingoeca rosetta DASH | Q | Y | L | I | D | W | 3 |
| Selaginella moellendorffii | Plant |  | [EFJ32286.1](https://www.ncbi.nlm.nih.gov/protein/EFJ32286.1?report=genbank&log$=protalign&blast_rank=1&RID=RBHTBHP2014) | Selaginella moellendorffii PHR | - | H | - | - | - | - |  |
| Setaria italica | Plant |  | [XP_004983328.1](https://www.ncbi.nlm.nih.gov/protein/XP_004983328.1?report=genbank&log$=protalign&blast_rank=1&RID=RBHRNGFA014) | Setaria italica CPDII | M | S | - | - | - | - | 2 |
| Shewanella algae | Gammaproteobacteria |  | [WP_044733894.1](https://www.ncbi.nlm.nih.gov/protein/WP_044733894.1?report=genbank&log$=protalign&blast_rank=1&RID=RBHGVS6N015) | Shewanella algae | M | W | L | V | D | W | 3 |
| Shinella sp. | Alphaproteobacteria |  | PZU23809.1 | Shinella | M | W | L | I | D | W |  |
| Siccationidurans arizonensis | Bacteroidetes | CRY-DASH | [WP_092670731.1](https://www.ncbi.nlm.nih.gov/protein/WP_092670731.1?report=genbank&log$=protalign&blast_rank=1&RID=KWAUY314015) | Siccationidurans arizonensis CD | Q | Y | H | Q | D | W | 2 |
| Solanum lycopersicum | Plant | Cry2 | [NP_001234245.1](https://www.ncbi.nlm.nih.gov/protein/NP_001234245.1?report=genbank&log$=protalign&blast_rank=1&RID=RBHF06T5014) | Solanum lycopersicum Cry2 | V | Y | L | L | P | W | 3 |
| Sorangiineae bacterium NIC37A_2 | Deltaproteobacteria |  | [OQX68931.1](https://www.ncbi.nlm.nih.gov/protein/OQX68931.1?report=genbank&log$=prottop&blast_rank=1&RID=KWAMM527014) | Sorangiineae bacterium | M | W | L | T | D | P | 1 |
| Sorghum bicolor | Plant | Cry | [XP_002436988.1](https://www.ncbi.nlm.nih.gov/protein/XP_002436988.1?report=genbank&log$=protalign&blast_rank=1&RID=RBHCKX1H014) | Sorghum bicolor Cry1 | V | Y | Q | I | P | W | 3 |
| Spodoptera exigua | Moth | Cry | [ADY17887.1](https://www.ncbi.nlm.nih.gov/protein/ADY17887.1?report=genbank&log$=protalign&blast_rank=1&RID=RBHA3900014) | Spodoptera exigua Cry | N | W | W | L | S | W | 4 |
| Spongiibacter sp. IMCC21906 | Gammaproteobacteria |  | [WP_047010836.1](https://www.ncbi.nlm.nih.gov/protein/WP_047010836.1?report=genbank&log$=protalign&blast_rank=1&RID=RBH76HSX015) | Spongiibacter sp. PL | M | W | H | I | D | W | 3 |
| Stanieria sp. NIES-3757 | Cyanobacteria | CRY-DASH | WP_096384486.1 | Stanieria sp. NIES3757 CD | Q | Y | G | I | N | W | 3 |
| Suberites domuncula | Sponge | Cry2 | [CAZ66367.1](https://www.ncbi.nlm.nih.gov/protein/CAZ66367.1?report=genbank&log$=protalign&blast_rank=1&RID=RBH3TEP7014) | Suberites domuncula CRY | Q | Q | W | I | S | W | 4 |
| Synechococcus PCC 7335 | Cyanobacteria | CRY-DASH | [WP_006456663.1](https://www.ncbi.nlm.nih.gov/protein/WP_006456663.1?report=genbank&log$=prottop&blast_rank=1&RID=RBGZYGAP015) | Synechococcus sp. PCC 7335 CD | Q | Y | G | I | D | W | 3 |
| Synechocystis PCC 6803 | Cyanobacteria |  | WP_014407099.1 | Synechocystis PCC 6083 | M | W | I | L | N | W |  |
| Synechocystis PCC 6803 | Cyanobacteria | CRY-DASH | WP_014407097.1 | Synechocystis PCC 6083 CD | Q | Y | G | I | D | W |  |
| [Syntrophorhabdus aromaticivorans](https://species.wikimedia.org/wiki/Syntrophorhabdus_aromaticivorans) | Deltaproteobacteria |  | [WP_028895847.1](https://www.ncbi.nlm.nih.gov/protein/WP_028895847.1?report=genbank&log$=protalign&blast_rank=1&RID=RBGYDWZT014) | Syntrophorhabdus aromaticivorans | M | K | - | - | - | - | 2 |
| Tetrahymena thermophila SB210 | Eukaryota |  | [XP_001014668.1](https://www.ncbi.nlm.nih.gov/protein/XP_001014668.1?report=genbank&log$=protalign&blast_rank=1&RID=RBGWBAYA015) | Tetrahymena thermophila | M | W | L | I | D | W | 3 |
| Tetraodon nigroviridis | Fish |  | [CAG04151.1](https://www.ncbi.nlm.nih.gov/protein/CAG04151.1?report=genbank&log$=protalign&blast_rank=1&RID=RBGT4W52014) | Tetraodon nigroviridis | H | W | W | I | G | W | 4 |
| Thalassiosira pseudonana CCMP1335 | Marine plankton diatom | (6-4) PL | XP_002291144.1 | Thalassiosira pseudonana 64 | H | W | W | Q | S | W | 4 |
| Thalassiosira pseudonana CCMP1335 | Marine plankton diatom | CRY-DASH | [XP_002291325.1](https://www.ncbi.nlm.nih.gov/protein/XP_002291325.1?report=genbank&log$=protalign&blast_rank=1&RID=RB3DNAH2014) | Thalassiosira pseudonana CD | Q | S | N | I | D | W | 3 |
| Thalassiosira pseudonana CCMP1335 | Marine plankton diatom | CPD II | [XP_002291031.1](https://www.ncbi.nlm.nih.gov/protein/XP_002291031.1?report=genbank&log$=protalign&blast_rank=1&RID=RB3BV1XR014) | Thalassiosira pseudonana CPDII | M | S | - | - | - | - | 2 |
| Thaumarchaeota archaeon | Archaea |  | [PHY04941.1](https://www.ncbi.nlm.nih.gov/protein/PHY04941.1?report=genbank&log$=protalign&blast_rank=1&RID=RBGR69F4015) | Thaumarchaeota archaeon | M | W | H | I | D | W | 3 |
| Thermocrinis minervae | Gram negative aquificales |  | [WP_079654270.1](https://www.ncbi.nlm.nih.gov/protein/WP_079654270.1?report=genbank&log$=protalign&blast_rank=1&RID=RBGMVC7V014) | Thermocrinis minervae | M | E | - | - | - | - | 2 |
| Thermoflexibacter ruber | FCB Bacteria | CRY-DASH | [WP_091539606.1](https://www.ncbi.nlm.nih.gov/protein/WP_091539606.1?report=genbank&log$=protalign&blast_rank=2&RID=RBGHZ600015) | Thermoflexibacter ruber | Q | Y | K | V | N | W | 2 |
| Thermoplasmatales archaeon SW_10_69_26 | Archaea |  | [PSG96639.1](https://www.ncbi.nlm.nih.gov/protein/PSG96639.1?report=genbank&log$=protalign&blast_rank=1&RID=RBGG0DZ2014) | Thermoplasmatales archaeon SW | M | W | L | L | P | W | 3 |
| Trypanosoma brucei brucei TREU927 | Parasite | FCPD | [XP_823319.1](https://www.ncbi.nlm.nih.gov/protein/XP_823319.1?report=genbank&log$=protalign&blast_rank=1&RID=RBGDB6Y8014) | Trypanosoma brucei FCPD | M | W | F | V | D | W | 3 |
| V[errucomicrobia bacterium](https://www.ncbi.nlm.nih.gov/Taxonomy/Browser/wwwtax.cgi?mode=Info&id=2026799) | Verrucomicrobia |  | RCL37293.1 | Verrucomicrobia bacterium | M | W | L | I | D | Y |  |
| Verticillium alfalfae VaMs.102 | Fungi |  | [P_002999933.1](https://www.ncbi.nlm.nih.gov/protein/XP_002999933.1?report=genbank&log$=protalign&blast_rank=1&RID=RBG7P1H9015) | Verticillium alfalfae VaMs.102 | M | F | L | I | D | W | 3 |
| Vibrio cholerae | Gammaproteobacteria | CD | [WP_000037642.1](https://www.ncbi.nlm.nih.gov/protein/WP_000037642.1?report=genbank&log$=protalign&blast_rank=1&RID=RBG4M2CN015) | Vibrio cholerae | Q | Y | G | L | D | W | 2 |
| Vitis vinifera | Plant | PHR2 | [XP_002270248.1](https://www.ncbi.nlm.nih.gov/protein/XP_002270248.1?report=genbank&log$=protalign&blast_rank=1&RID=RBG1F2ER015) | Vitis vinifera PHR2 | - | Y | - | - | - | - |  |
| [Xanthomonas axonopodis Xac29-1](https://www.ncbi.nlm.nih.gov/Taxonomy/Browser/wwwtax.cgi?mode=Info&id=1304892) | Gammaproteobacteria | CPD | WP_011050942.1 | Xanthomonas axonopodis CPD | M | W | R | I | H | W | 3 |
| Xenopus laevis | Eukaryote | (6-4) PL | [NP_001081421.1](https://www.ncbi.nlm.nih.gov/protein/NP_001081421.1?report=genbank&log$=protalign&blast_rank=1&RID=RB409NY8014) | Xenopus laevis 64PL | H | W | W | I | S | W | 4 |
| Xenopus laevis | Eukaryote | CPD II | [NP_001089127.1](https://www.ncbi.nlm.nih.gov/protein/NP_001089127.1?report=genbank&log$=protalign&blast_rank=1&RID=RB3YJZK7014) | Xenopus laevis CPD II | M | S | - | - | - | - | 2 |
| Xenopus laevis | Eukaryote | CRY-DASH | [NP_001084438.1](https://www.ncbi.nlm.nih.gov/protein/NP_001084438.1?report=genbank&log$=protalign&blast_rank=1&RID=RB434U0E014) | Xenopus laevis DASH | Q | Y | G | I | D | W | 3 |
| Xenopus tropicalis | Eukaryote | Cry4 | [BAO09600.1](https://www.ncbi.nlm.nih.gov/protein/BAO09600.1?report=genbank&log$=protalign&blast_rank=1&RID=RB41X2GF014) | Xenopus tropicalis CRY4 | H | W | W | I | S | W | 4 |
| Zea mays | Plant | PHR2 | [NP_001131008.1](https://www.ncbi.nlm.nih.gov/protein/NP_001131008.1?report=genbank&log$=protalign&blast_rank=1&RID=RB45TXY7014) | Zea mays PHR2 | - | Y | - | - | - | - |  |
| candidate division Zixibacteria bacterium HGW-Zixibacteria-1 | FCB group bacteria |  | [PKK83216.1](https://www.ncbi.nlm.nih.gov/protein/PKK83216.1?report=genbank&log$=protalign&blast_rank=1&RID=RB4973BU014) | Zixibacteria bacterium | M | A | - | - | - | - | 2 |
| Zymoseptoria tritici IPO323 | Fungi |  | [XP_003857369.1](https://www.ncbi.nlm.nih.gov/protein/XP_003857369.1?report=genbank&log$=protalign&blast_rank=1&RID=RB3R68CK015) | Zymoseptoria tritici 64PL | H | W | Y | I | S | W | 3 |
| Zymoseptoria tritici IPO323 | Fungi | CD | [XP_003853581.1](https://www.ncbi.nlm.nih.gov/protein/XP_003853581.1?report=genbank&log$=protalign&blast_rank=1&RID=RB3N3AC4014) | Zymoseptoria tritici CD | Q | Y | E | I | D | W | 2 |
| Zymoseptoria tritici IPO323 | Fungi | CPD | [XP_003847594.1](https://www.ncbi.nlm.nih.gov/protein/XP_003847594.1?report=genbank&log$=protalign&blast_rank=1&RID=RB3J1K99014) | Zymoseptoria tritici CPD | M | - | L | I | D | Y | 2 |
| Zymoseptoria tritici IPO323 | Fungi |  | [XP_003857772.1](https://www.ncbi.nlm.nih.gov/protein/XP_003857772.1?report=genbank&log$=protalign&blast_rank=1&RID=RB3KHFF9014) | Zymoseptoria tritici CPDI | M | F | G | V | D | W | 3 |
